# Real-Life Gait Performance as a Digital Biomarker for Motor Fluctuations: The Parkinson@Home Validation Study

**DOI:** 10.2196/19068

**Published:** 2020-10-09

**Authors:** Luc JW Evers, Yordan P Raykov, Jesse H Krijthe, Ana Lígia Silva de Lima, Reham Badawy, Kasper Claes, Tom M Heskes, Max A Little, Marjan J Meinders, Bastiaan R Bloem

**Affiliations:** 1 Center of Expertise for Parkinson and Movement Disorders, department of Neurology Donders Institute for Brain, Cognition and Behaviour Radboud University Medical Center Nijmegen Netherlands; 2 Institute for Computing and Information Sciences Radboud University Nijmegen Netherlands; 3 Department of Mathematics School of Engineering and Applied Sciences Aston University Birmingham United Kingdom; 4 School of Computer Science University of Birmingham Birmingham United Kingdom; 5 UCB Pharma Brussels Belgium; 6 Scientific Center for Quality of Healthcare (IQ healthcare) Radboud Institute for Health Sciences Radboud University Medical Center Nijmegen Netherlands

**Keywords:** digital biomarkers, remote patient monitoring, wearable sensors, real-life gait, Parkinson disease, biomarker, patient monitoring, wearables, gait

## Abstract

**Background:**

Wearable sensors have been used successfully to characterize bradykinetic gait in patients with Parkinson disease (PD), but most studies to date have been conducted in highly controlled laboratory environments.

**Objective:**

This paper aims to assess whether sensor-based analysis of real-life gait can be used to objectively and remotely monitor motor fluctuations in PD.

**Methods:**

The Parkinson@Home validation study provides a new reference data set for the development of digital biomarkers to monitor persons with PD in daily life. Specifically, a group of 25 patients with PD with motor fluctuations and 25 age-matched controls performed unscripted daily activities in and around their homes for at least one hour while being recorded on video. Patients with PD did this twice: once after overnight withdrawal of dopaminergic medication and again 1 hour after medication intake. Participants wore sensors on both wrists and ankles, on the lower back, and in the front pants pocket, capturing movement and contextual data. Gait segments of 25 seconds were extracted from accelerometer signals based on manual video annotations. The power spectral density of each segment and device was estimated using Welch’s method, from which the total power in the 0.5- to 10-Hz band, width of the dominant frequency, and cadence were derived. The ability to discriminate between before and after medication intake and between patients with PD and controls was evaluated using leave-one-subject-out nested cross-validation.

**Results:**

From 18 patients with PD (11 men; median age 65 years) and 24 controls (13 men; median age 68 years), ≥10 gait segments were available. Using logistic LASSO (least absolute shrinkage and selection operator) regression, we classified whether the unscripted gait segments occurred before or after medication intake, with mean area under the receiver operator curves (AUCs) varying between 0.70 (ankle of least affected side, 95% CI 0.60-0.81) and 0.82 (ankle of most affected side, 95% CI 0.72-0.92) across sensor locations. Combining all sensor locations did not significantly improve classification (AUC 0.84, 95% CI 0.75-0.93). Of all signal properties, the total power in the 0.5- to 10-Hz band was most responsive to dopaminergic medication. Discriminating between patients with PD and controls was generally more difficult (AUC of all sensor locations combined: 0.76, 95% CI 0.62-0.90). The video recordings revealed that the positioning of the hands during real-life gait had a substantial impact on the power spectral density of both the wrist and pants pocket sensor.

**Conclusions:**

We present a new video-referenced data set that includes unscripted activities in and around the participants’ homes. Using this data set, we show the feasibility of using sensor-based analysis of real-life gait to monitor motor fluctuations with a single sensor location. Future work may assess the value of contextual sensors to control for real-world confounders.

## Introduction

### Background

The core treatment of patients with Parkinson disease (PD) is symptomatic and consists of dopamine replacement therapy. Although motor symptoms such as bradykinesia and rigidity can be well controlled in early disease stages, most patients experience motor fluctuations in symptom severity after a few years of treatment with levodopa [1]. The pattern of these motor fluctuations varies between patients and may include wearing off, unpredictable off phases, peak-dose dyskinesias, and diphasic dyskinesias. Therefore, optimal management of motor fluctuations requires a highly personalized approach. The current evaluation of motor fluctuations involves paper diaries, such as the Hauser diary, which are burdensome to complete and which demonstrate reduced compliance when used for more than three days [2]. Moreover, patients are not always able to accurately recognize their own symptoms (eg, dyskinesia is often confused with tremor and vice versa). A more objective and unobtrusive way to assess motor fluctuations over longer periods of time in daily life could shed light on the real-world presentation of PD and benefit both individual patient care and follow-up in clinical trials.

Wearable movement sensors could potentially address this need. It is important to distinguish between active monitoring (ie, the analysis of specific tasks, such as the timed up and go [TUG] test [3]) and passive monitoring (ie, the analysis of natural behavior of daily living). Although active monitoring has received by far the most attention [4,5], this approach is susceptible to attrition in patient compliance [6]. By contrast, excellent long-term compliance can be achieved when patients are only asked to wear an unobtrusive sensor, such as a smartwatch, as they go about their daily activities [7]. Passive monitoring can also provide more continuous insights into symptom fluctuations throughout the day. However, these benefits come at a cost: it is challenging to develop algorithms that can cope with the large variation in signals encountered in daily life, of which only a small proportion may be explained by PD-related impairments. Since obtaining accurate labels in daily life environments is difficult and costly, most of the currently available reference data sets have been collected in the lab and involved patients performing a standardized set of tasks and activities. Algorithms trained on these data sets are unlikely to perform well in real-life settings [8]. Some studies have simulated a home environment in the lab and included unscripted behavior [9,10]. Although this is more realistic, all data are still collected in the same environment, so the expected variation in activities and symptom severity is much reduced compared with real life. Consequently, the real-life performance of new sensor-based methods to assess motor fluctuations remains largely unknown.

Another challenge is finding the best strategy to derive meaningful and interpretable outcome measures from daily life sensor data. One approach is to detect and quantify a specific behavior that probes the presence of PD symptoms. Gait is a promising candidate in this regard for three reasons. First, gait is highly stereotypic, allowing for accurate detection in daily life signals. Second, gait becomes abnormal even in very early stages of the disease, likely already in the prodromal phase [11], and gait impairments worsen as the disease progresses [12,13]. Recording and analyzing gait is therefore useful across a wide spectrum of disease severities. Third, even among patients with PD without freezing of gait, the gait pattern changes in response to dopaminergic medication. Specifically, the step length and arm swing are reduced in the off state, which is known as bradykinetic gait [14,15]. However, these findings are based on data collected during standardized tasks (typically the TUG test) in highly controlled laboratory environments. It is not self-evident that these effects directly translate to daily life [16]. Various behavioral and environmental factors may influence gait in daily life. In addition, patients with PD show different gait patterns when they are aware that they are being evaluated (partially a Hawthorne effect and partially the phenomenon of *kinesia paradoxa*, the sudden transient ability of patients with PD to perform a task due to increased alertness or arousal) [17,18]. Indeed, gait patterns as measured in the lab and in real life (using accelerometry) are only weakly correlated [19,20].

Empirical evidence that underlines the value of analyzing real-life gait in PD is emerging. This approach may discriminate better than lab-based gait analysis between patients with PD and controls [19]. Similarly, real-life gait variability, derived from an accelerometer worn on the lower back, may predict the time to first fall better than various in-lab measurements, including gait speed in the off state [21]. In addition, a pilot study showed that at-home gait speed, measured using a radio wave–based monitor, correlates with PD severity [22]. However, studies that have examined how on-off changes are reflected in real-life gait patterns are scarce. Moore et al [23] suggested that step length derived from an ankle sensor could be used to monitor on-off changes in real life but illustrated this in only one patient. Sama et al [24] proposed that the energy in the 0- to 10-Hz frequency band during gait, obtained using a waist-worn sensor, could predict the patient’s motor state. When tested in daily life, the algorithm’s predictions demonstrated high agreement with on-off diaries completed by the patients [25]. We conclude that analyzing real-life gait to monitor motor fluctuations has thus far shown promising results, but developments remain in the early stages. This is reflected by the lack of consensus on the best sensor location [26]. The field would benefit from an objective comparison of commonly used sensor locations on labelled data collected in the patients’ home environments.

### Study Objective

In the Parkinson@Home validation study, we collected data from multiple wearable sensors during unscripted activities in and around the participants’ homes and recorded the activities on video. The objective of this publication is twofold. First, we aim to describe this new reference data set, which will be made available to the research community in collaboration with the Michael J Fox Foundation. Second, using this data set, we aim to assess whether real-life gait analysis can be used to obtain a digital biomarker for motor fluctuations in PD. Specifically, we will assess how well various sensor locations can discriminate between gait performance before and after intake of dopaminergic medication and explore which factors complicate gait analysis in daily life.

## Methods

### Study Sample

We included a group of 25 patients with PD and 25 age-matched participants without PD (controls). Patients were recruited using various strategies, including advertisements in the Dutch Parkinson Patient Association’s magazine and on social media, visits to support groups, and through physiotherapists specialized in the treatment of PD. Controls were recruited from partners and acquaintances of the participating patients and by advertisements on social media. The inclusion criteria were (1) aged ≥30 years, (2) in possession of a smartphone running on Android 4.4 or higher, and (3) living within travelling distance from the study center. Additional inclusion criteria for the PD group were (1) Parkinson disease diagnosed by a neurologist, (2) currently using levodopa and/or a dopamine agonist, (3) experiencing at least slight motor fluctuations (Movement Disorders Society Unified Parkinson’s Disease Rating Scale [MDS-UPDRS] part IV item 4.3 ≥1), and (4) experiencing at least some Parkinson-related gait impairments (MDS-UPDRS part II item 2.12 ≥1 and/or item 2.13 ≥1). The exclusion criteria were (1) any type of advanced treatment (deep brain stimulation or intestinal levodopa or apomorphine infusion) and (2) psychiatric or cognitive impairments that may hinder successful completion of the study protocol (based on judgement of the assessor running the recruitment). We did not exclude patients with PD or controls who used assistive devices or reported other medical problems affecting their movements. We refer to Multimedia Appendix 1 for a flow diagram of the inclusion procedure.

### Data Collection Procedure

Data were collected during single visits to the participants’ homes from July 2017 to July 2018. A visit consisted of 2 parts: an unscripted free-living part and a standardized clinical assessment. In the PD group, both parts were conducted twice during the same visit, once in the morning after overnight withdrawal of dopaminergic medication (premedication) and once after intake of the patient’s prescribed dopaminergic medication (postmedication).

During the free-living part, the assessors encouraged the participants to perform habitual activities in and around their house for at least one hour. In order to capture natural behavior, there was no script for this part of the visit. Instead, the assessors used a checklist to ensure that essential behaviors were captured, such as doing normal morning routines, preparing and having breakfast, walking indoors, walking in the neighborhood, walking up and down the stairs, sitting down, standing up, and doing some household chores. It should be noted that the participants’ normal routines were leading; for example, if participants hardly did any household chores themselves, we did not ask them to do so during the visit.

The clinical assessments were also conducted in the participants’ homes and included the TUG test, the Abnormal Involuntary Movement Scale, and the complete MDS-UPDRS, except for the self-reported items of part I and II, which were completed through an online survey after the visit. In the PD group, the clinical assessments were performed before and after medication intake, except for part I (assessor-rated items) and part IV of the MDS-UPDRS, which were only performed after medication intake. In addition, both the patient and the assessor scored the disease state (as off, on without dyskinesia, or on with dyskinesia) for every 30-minute epoch after medication intake. All assessments were conducted by a single assessor who had received appropriate prior training. Multimedia Appendix 1 provides an overview of the events during the home visits.

During the full duration of the visit, participants wore lightweight sensors on both wrists, both ankles, the lower back, and in the front pants pocket, containing movement, contextual, and physiological sensors (Table 1). Except for the smartphone, all devices were attached using adjustable straps. In addition, the entire visits were recorded on video using a handheld high-definition video recorder by one of the assessors following the participant. To put the patient at ease and make the setting more naturalistic, the other assessor helped to comfort the patient and shift the focus from being recorded to performing habitual activities together. To allow for time synchronization between all sensors and the video recordings, all devices were triggered (hit simultaneously against the table 10 times) in front of the camera at the beginning and end of the recordings.

**Table 1:** Overview of the wearable sensors used during the study visits.

Device	Locations	Collected sensor data
Gait Up Physilog 4^a^	Both ankles, both wrists, lower back (strap around waist)	Accelerometer, gyroscope, magnetometer, barometer
Android Wear smartwatch^b^	Wrist (PD^c^ group: self-reported most affected side; control group: most comfortable side)	Accelerometer, gyroscope, barometer, light
Android smartphone^d^	Pants pocket (same side as Android Wear smartwatch)	Accelerometer, magnetometer, light, proximity, GPS, Wi-Fi, and cellular networks
Empatica E4^e^	Wrist (opposite wrist to Android Wear smartwatch)	GSR^f^, PPG^g^, skin temperature, accelerometer

^a^Gait Up SA.

^b^Motorola Moto 360 Sport (Motorola Inc) with custom application collecting raw sensor data.

^c^PD: Parkinson disease.

^d^Various models with the HopkinsPD app collecting raw sensor data.

^e^Empatica Inc.

^f^GSR: galvanic skin response.

^g^PPG: photoplethysmogram.

After the home visits, all participants continued to use a subset of the sensors (smartphone and smartwatch) for 2 weeks and completed symptom diaries as reference. As this part of the data set is not used in the current analyses, we refer to Multimedia Appendix 1 for a detailed description of the corresponding protocol.

The study protocol was approved by the local medical ethics committee (Commissie Mensgebonden Onderzoek, region Arnhem-Nijmegen, file number 2016-1776). All participants received verbal and written information about the study protocol and signed a consent form prior to participation, in line with the Declaration of Helsinki.

### Data Availability

The full data set as described in the “Methods” section, with the exception of the raw video recordings and absolute GPS coordinates, will be made available to the worldwide research community in collaboration with the Michael J Fox Foundation. To protect participant privacy, manual annotations are available from the video recordings, and the GPS data will be deidentified before sharing. A subset of the data is being used in the international Biomarker and Endpoint Assessment to Track Parkinson’s Disease (BEAT-PD) Challenge [27]. The full curated and deidentified data set will be released when this data challenge is completed. The specific data and analysis scripts that support the findings of this study are available from the corresponding author upon reasonable request.

### Data Processing

#### Video Annotations

To provide ground truth labels for algorithm development and validation, the video recordings were annotated by trained research assistants for (1) the protocol structure (ie, when the clinical assessments and free-living parts were performed), (2) the occurrence of general behaviors during the free-living parts (such as standing, walking, and sitting), and (3) the presence and severity of tremor and the presence and manifestations of freezing of gait during the free-living parts. Annotations from the last category were checked by a physician with experience in movement disorders. In addition, those items of the MDS-UPDRS part III that can be evaluated from video were assessed by a second, independent rater (physician with experience in movement disorders). The annotations were created using ELAN (The Language Archive), an open source program for creating annotations in video recordings [28]. For a detailed description of the video annotation protocol, we refer to Multimedia Appendix 1.

#### Sensor Data Preprocessing

In the current analysis, we used the triaxial accelerometer data (in m/s^2^) from all Physilog devices and the smartphone worn in the pants pocket. First, data were interpolated to a uniform sample rate of 120 Hz using piecewise cubic interpolation. Next, the effects of gravity were removed by applying an ℓ_1_ trend filter to each of the 3 axes separately (MATLAB implementation by Kim et al [29]). For each device, we used the 3 resulting dynamic acceleration signals, a_x_, a_y_, and a_z_, to compute the magnitude of dynamic acceleration (ie, the square root of [a_x_^2^ + a_y_^2^ + a_z_^2^]).

#### Frequency Analysis of Gait Segments

Various approaches have been used to quantify the gait pattern in patients with PD using accelerometer data. Some studies rely on the detection of the initial and final contact of the feet, from which temporal gait features such as the step and swing time can be derived [30]. Based on the exact sensor positioning and some assumptions derived from the biomechanics of gait, location-specific algorithms can be used to estimate spatial gait features. For example, having identified the initial and final contact, one can use the inverted pendulum model to estimate the step length using a sensor on the lower back [31]. Although this approach produces outcome measures that directly relate to the way gait is evaluated by clinicians, its location dependency complicates the comparison of different sensor locations. Additionally, detecting the initial and final contact is more challenging in real-life circumstances [32]. Other studies analyze the periodicity of the accelerometer signal during gait based on the power spectral density (PSD) or autocorrelation [21,33]. Since it does not rely on location-specific assumptions, we used this approach in our analysis.

The video annotations were used to locate periods of gait (defined as five or more consecutive steps) during the free-living parts. From these, we extracted nonoverlapping gait segments of equal length (3000 samples, corresponding to 25 seconds). This length was selected because prior research showed that using shorter free-living gait segments discriminated less well between patients with PD and controls [19] and in order to achieve sufficient resolution in the frequency domain. To be included in the analyses, participants needed to have at least 10 gait segments of 25 seconds. In addition, patients were required to have at least 5 segments before and 5 segments after medication intake. The PSD of each gait segment and sensor location was estimated using Welch’s method [34] (with, per segment, Hamming windows of 1024 samples with approximately 50% overlap). From each of the 6 sensor locations, we extracted 4 different signal properties, resulting in 24 features. We computed the total power in the 0.5- to 10-Hz interval, which captures practically all contributions from human gait [35]. In addition, we extracted the frequency, height, and width (at half the height) of the dominant peak in the PSD. The frequency of the dominant peak was used to derive the participants’ cadence (the method is described in Multimedia Appendix 1).

### Evaluation

For the main objective of this study, we compared real-life gait segments before and after medication intake. Since we expected that the relative differences within subjects are most relevant in this context, we normalized each feature using *z* scores based on each patient’s mean and standard deviation. In addition, we compared gait segments between patients with PD and controls. For these analyses, we rescaled each feature using its between-subject standard deviation in order to aid the comparison of effect sizes between features and to obtain a common scale for regularization (see “Classification”).

#### Individual Features

First, we examined the effect of medication intake on the individual features. To deal with the varying number of gait segments between participants, we used linear mixed effect models with the normalized features as dependent variables. For each feature, we estimated a fixed effect of the timing of the gait segment (premedication/postmedication) and random intercepts and slopes per patient. We used separate linear mixed effect models to estimate fixed effects of the group (patients with PD premedication, postmedication, or controls) and random intercepts per participant. Because we aim to show the magnitude and spread of the individual fixed effects rather than testing overarching hypotheses based on multiple comparisons, we report the unadjusted 95% confidence intervals of the estimated effects, as recommended by Gelman et al [36].

#### Classification

Next, we evaluated whether combinations of features could be used to predict whether a gait segment occurred before or after medication intake. For this, we used logistic LASSO (least absolute shrinkage and selection operator) regression with uniform prior class probabilities. To account for the varying number of gait segments per patient, we weighted each gait segment by the inverse of the number of gait segments per patient. We evaluated the performance using leave-one-subject-out nested cross-validation (CV), with the LASSO regularization hyperparameter being selected in the inner CV loops. The main performance measure was the mean area under the receiver operator curve (AUC). In addition, we evaluated the performance of logistic LASSO regression to predict whether a gait segment was from a patient with PD (premedication) or control. We used nested cross-validation for this as well, leaving 1 patient and 1 control out in each fold. To avoid information leakage, normalization was performed separately for each fold using only training data.

For both problems, we trained one classifier for each sensor location and one for all sensor locations combined. To prevent the patient with PD or control classifiers from learning the differences between the dominant and nondominant side, we ensured that the proportion of participants with measurements from the dominant side was always equal in the PD and control group. We tested whether the AUCs of the individual sensor locations were lower than the AUC of all sensor locations combined using the Wilcoxon matched pairs signed rank test (*P*<.05 considered statistically significant). The results of the individual comparisons were then used to test the overarching null hypothesis that using all sensor locations combined is superior to using any of the single sensor locations (we rejected this hypothesis if three or more individual comparisons were nonsignificant, which corresponds to a significance level α of approximately .01; for the calculation, see Multimedia Appendix 1).

Using the trained classifiers of the sensor location with the highest AUC, we constructed continuous scores that could serve as digital biomarkers for the response to medication intake (premedication/postmedication classifier) and PD gait impairment severity (PD/control classifier). For this, we used the classifiers’ decision values (linear combinations of feature values), which, in the case of logistic regression, correspond to the logarithm of the odds (logit) of the posterior class probabilities. We then examined their correlation with relevant clinical measures (Spearman ρ). Specifically, using the “best” classifier for predicting premedication and postmedication in the PD group, the patients’ mean changes in decision values after medication intake (corresponding to the patients’ mean log odds ratio) were correlated to the changes in the TUG score and the subtotal of the items related to mobility of the MDS-UPDRS part III (items 3.9, 3.10, 3.11, 3.12, and 3.13). Using the same sensor location’s classifier for predicting patient with PD or control, the patients’ mean decision values were correlated to the absolute TUG score, time since diagnosis, and the subtotal of the MDS-UPDRS part III mobility items. All analyses were performed in MATLAB 2018a (MathWorks).

#### Qualitative Evaluation

To explore which factors unrelated to PD may disturb gait analysis in real-world settings, we inspected individual patients and sensor locations in which the classifier performed worse than random classification in predicting premedication and postmedication (AUC <0.5). This was done by visually identifying change points in the PSDs and evaluating the corresponding video recordings for potential explanations. If an identified factor was also present in the video recordings of another patient, we evaluated whether it had a similar impact on the PSD (regardless of the patient’s AUC).

## Results

### Participant Characteristics

A minimum of 10 gait segments of 25 seconds were available in 18 patients with PD (median 46.5 segments, range 14-95) and 24 controls (median 31 segments, range 11-61). These participants’ demographic and clinical characteristics are presented in Table 2. Reasons for collecting an insufficient number of gait segments included rainy weather (n=4), a desire not to be filmed in the neighborhood (n=1), use of a wheelchair for longer distances (n=1), fatigue (n=1), and technical problems with the video recordings (n=1). The included patients did not differ substantially from the excluded patients in terms of disease severity (Multimedia Appendix 1). In 9 participants, there were technical problems with the sensor worn on the lower back. Therefore, we excluded this sensor location from the analyses combining multiple sensor locations (we refer to the Multimedia Appendix 1 for the results of the 33 patients for whom the lower back sensor data were available). In addition, technical problems caused data loss for 1 ankle sensor in 1 participant; this participant (control) was excluded from the analyses combining multiple sensor locations. The distribution of the length of the analyzed gait segments was similar among patients with PD (premedication and postmedication) and controls (Multimedia Appendix 1). The experiences of participants during the home visit collected by the online exit survey are included in Multimedia Appendix 1.

**Table 2:** Demographics and clinical characteristics of patients included in the analyses.

Characteristic			Patients with PD^a^ (n=18)		Controls (n=24)
Age (years), median (IQR)			65.0 (60.5-69.0)		67.5 (55.0-70.0)
Gender (men), n (%)			11 (61)		13 (54)
**Most affected side^b^ and hand dominance, n (%)**					
	Most affected=dominant	8 (44)		N/A^c^	
	Most affected=nondominant	8 (44)		N/A	
	Mixed handedness	2 (11)		N/A	
Time since diagnosis of PD (years), median (IQR)			6.5 (4.8-10.3)		N/A
**Use of dopaminergic medication, n (%)**					
	Levodopa only	6 (33)		N/A	
	Levodopa and dopamine agonist	10 (55)		N/A	
	Levodopa and MAO-B^d^ inhibitor	1 (6)		N/A	
	Levodopa, dopamine agonist, and MAO-B inhibitor	1 (6)		N/A	
**Hoehn & Yahr stage, n (%)**					
	Stage 1	1 (6)		N/A	
	Stage 2	13 (72)		N/A	
	Stage 3	4 (22)		N/A	
**MDS-UPDRS^e^ (scores on subscales), median (IQR)**					
	Part I (scale range: 0 to 52)	9.5 (7.8-15.0)		3.0 (0.3-4.0)	
	Part II (scale range: 0 to 52)	11.0 (8.5-15.3)		0.0 (0.0-0.0)^f^	
	Part III (off state) (scale range: 0 to 132)	41.5 (31.5-57.8)		6.5 (4.3-11.0)	
	Part III (on state) (scale range: 0 to 132)	28.0 (18.5-38.0)		N/A	
	Part IV^g^ (scale range: 0 to 24)	6.0 (4.5-9.3)		N/A	
**AIMS^h^ (scale range: 0 to 40), n (%)**					
	0	13 (72)		20 (83)	
	1-3	2 (11)		3 (13)	
	>3	3 (17)		1 (4)^i^	
**TUG^j^ (median of 4 trials, in seconds), median (IQR)**					
	Off state	12.0 (11.3-13.7)		10.0 (9.3-10.8)	
	On state	11.4 (9.7-12.4)		N/A	
**Falls in last 12 months, n (%)**					
	0	10 (56)		20 (83)	
	1-2	8 (44)		4 (17)	
**Freezing episodes in last month, n (%)**					
	0	13 (72)		N/A	
	1 or more	5 (28)		N/A	

^a^PD: Parksinson disease.

^b^Most affected side: side where the PD symptoms are most severe, as reported by the patients.

^c^N/A: not applicable.

^d^MAO-B: monamine oxidase B.

^e^MDS-UPDRS: Movement Disorders Society Unified Parkinson’s Disease Rating Scale.

^f^1 missing value.

^g^Specific to PD: side effects of dopaminergic medication.

^h^AIMS: Abnormal Involuntary Movement Scale.

^i^This participant demonstrated facial synkinesis during the assessment.

^j^TUG: timed up and go test.

### Comparison of Before and After Medication Intake

**Figure 1:**
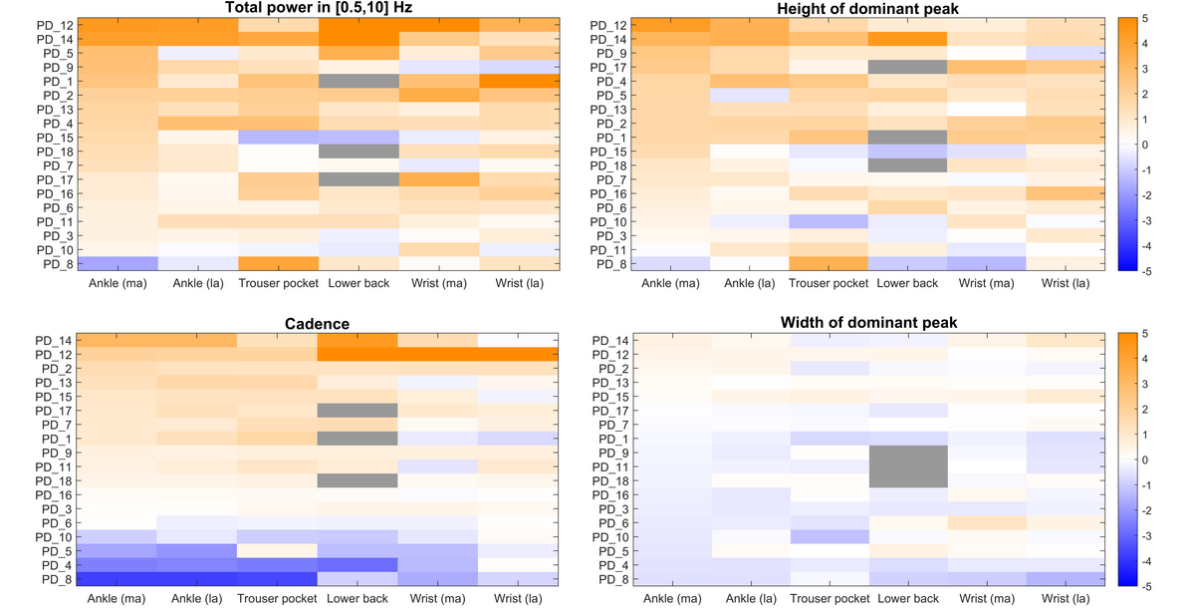
Changes in the 4 signal properties after medication intake, expressed in z scores (color bar). The x-axis displays the various sensor locations; the y-axis shows all individual patients (sorted on the values of the ankle sensor of the most affected side). The figure highlights that the total power and height of the dominant peak increase after medication intake in most patients (with considerable variation between sensor locations), the cadence increases in some but decreases in others (with high agreement between sensor locations), and the width of the dominant peak does not change considerably. Grey areas indicate missing data. la: least affected; ma: most affected; PD: Parkinson disease.

**Figure 2:**
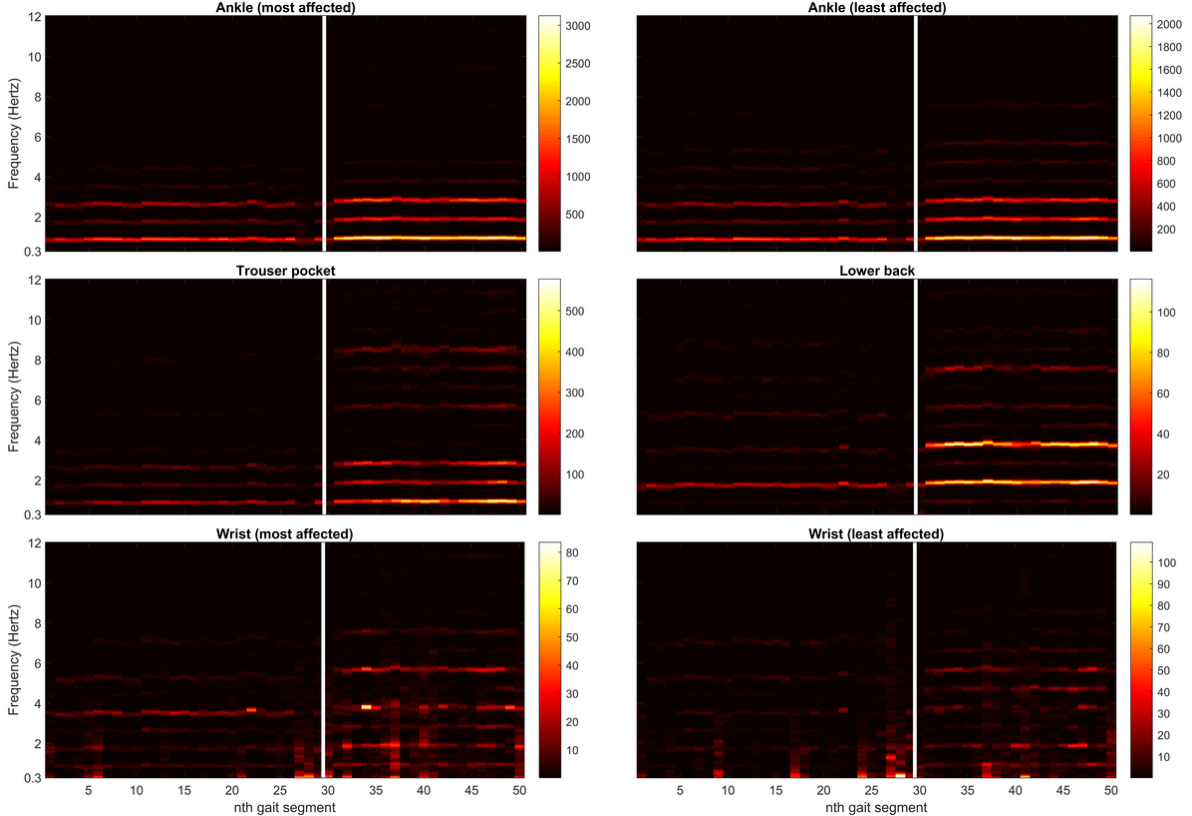
Visualization of the PSD of one of the patients who shows a clear response after medication intake (PD_14). x-axis: nth gait segment of 25 seconds (the white line indicates intake of dopaminergic medication). y-axis: frequency (Hertz). Color bar: PSD in (m/s^2^)^2^/Hz. PD: Parkinson disease; PSD: power spectral density.

**Figure 3:**
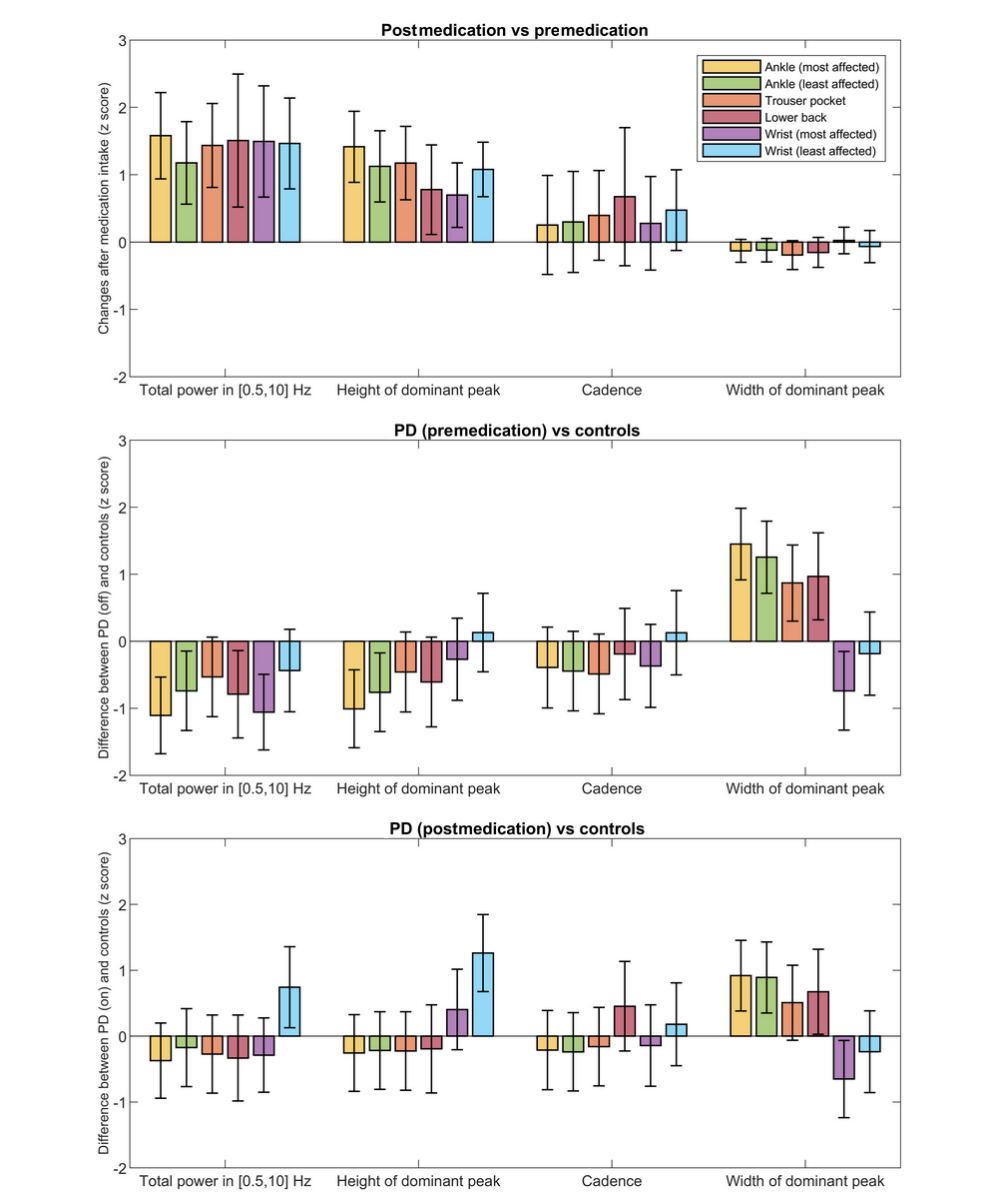
Top: changes after medication intake on a group level (mean and 95% CI). Middle: differences between patients with PD (premedication) and controls (mean and 95% CI, positive means higher in patients). Bottom: differences between patients with PD (postmedication) and controls (mean and 95% CI, positive means higher in patients). All estimates are based on linear mixed effects models for each sensor location and signal property. PD: Parkinson disease.

Figure 1 shows all individual changes in the signal properties after medication intake. For illustration, Figure 2 displays the power spectral density of one of the patients who demonstrated a clear response to medication intake (PD_14). At the group level, the total power and height of the dominant peak increased in all sensor locations after medication intake, whereas the width of the dominant peak did not change significantly in any sensor location (Figure 3). Cadence increased in some patients but markedly decreased in others, resulting in a nonsignificant change at the group level for all sensor locations (Figure 3). Because of the high correlation between changes in the height of the dominant peak and total power (mean Pearson *r* of 0.80) and the clearer response of the latter across the sensor locations, the height was not included in subsequent analyses.

**Figure 4:**
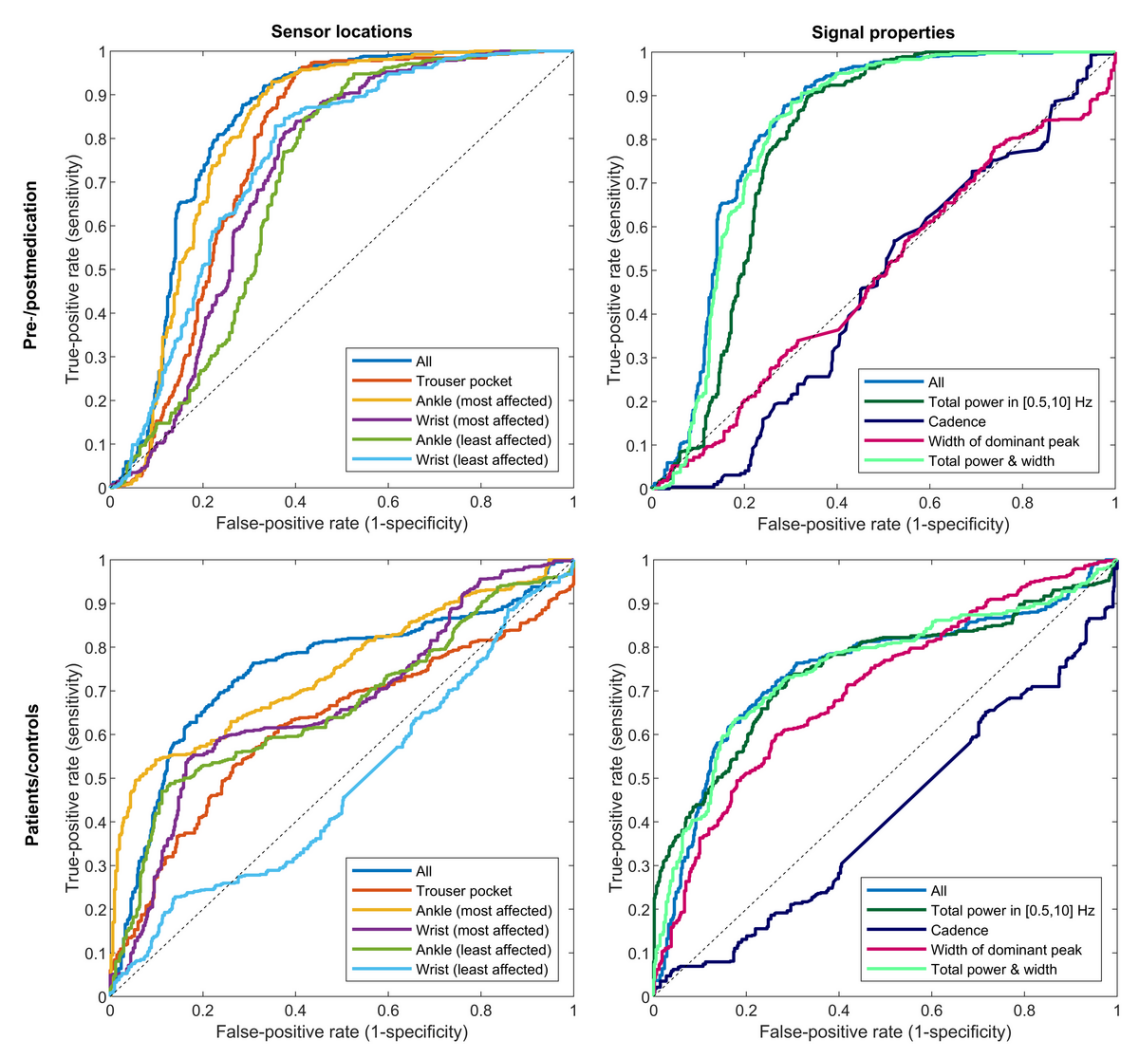
Receiver operating characteristic curves of the logistic classifiers, averaged over the cross-validation folds. Top half: premedication and postmedication classification. Bottom half: patients and controls classification. Left half: comparison between different sensor locations. Right half: comparison between different signal properties, based on all sensor locations combined.

Figure 4 displays the receiver operator curves (ROCs) of the logistic classifiers trained using the various sensor locations and signal properties. The mean AUC for all sensor locations and signal properties combined was 0.84 (95% CI 0.75-0.93). The mean AUCs of the ankle of the most affected side (0.82, 95% CI 0.72-0.92), the wrist of the most affected side (0.76, 95% CI 0.66-0.87), and the wrist of the least affected side (0.79, 95% CI 0.69-0.88) were not significantly worse than using all sensor locations combined. Based on this, we reject the overarching hypothesis that using all sensor locations combined is superior to using any of the single sensor locations. Of the signal properties, the total power was most informative to discriminate between before and after medication intake, with a mean AUC of 0.80 (95% CI 0.69-0.91). The mean AUC of the width of the dominant peak (0.51, 95% CI 0.45-0.58) and cadence (0.55, 95% CI 0.43-0.66) were by themselves not significantly different from 0.5 (random classifier), although the width of the dominant peak resulted in a modest (but statistically significant) improvement in AUC when combined with the total power (Figure 4). The AUC and balanced accuracy (based on an equal class prior) of all locations and signal properties are presented in Table 3. Multimedia Appendix 1 includes a sensitivity analysis on the effects of the choice of per-subject normalization.

Both the TUG (–1.51, 95% CI –2.29 to –0.73) and the subtotal of the MDS-UPDRS part III mobility items (–1.31, 95% CI –2.13 to –0.48) decreased after medication intake. However, the patients’ mean changes in decision values, obtained from the classifier based on the ankle sensor of the most affected side, did not correlate with changes in the TUG (ρ=–0.02, 95% CI –0.49 to 0.45), nor with changes in the subtotal of the MDS-UPDRS part III mobility items (ρ=0.11, 95% CI –0.38 to 0.45).

Based on the video recordings, freezing of gait (FOG) was observed in 2 patients (PD_1 and PD_4). However, no FOG episodes occurred during any of the gait segments of at least 25 seconds, so FOG is not expected to have influenced the observed changes.

**Table 3:** Performance of the logistic classifiers (mean, SE 1.96 over the cross-validation folds). Accuracies are based on the optimal classifier for each fold with equal misclassification costs and equal class prior (also referred to as balanced accuracy).

Feature set		Premedication/postmedication, mean (95% CI)				Patients with PD^a^ (premedication) versus controls, mean (95% CI)			
		AUC^b^		Accuracy		AUC		Accuracy	
All		0.84 (0.75-0.93)		0.79 (0.71-0.87)		0.76 (0.62-0.90)		0.72 (0.61-0.83)	
**Sensor locations**									
	Pants pocket		0.78 (0.67-0.89)		0.75 (0.67-0.83)		0.62 (0.46-0.78)		0.63 (0.51-0.74)
	Ankle (most affected)		0.82 (0.72-0.92)		0.77 (0.71-0.84)		0.74 (0.58-0.90)		0.66 (0.54-0.79)
	Wrist (most affected)		0.76 (0.66-0.87)		0.72 (0.64-0.81)		0.75 (0.62-0.88)		0.57 (0.50-0.65)
	Ankle (least affected)		0.70 (0.60-0.81)		0.68 (0.60-0.76)		0.70 (0.53-0.86)		0.62 (0.50-0.74)
	Wrist (least affected)		0.79 (0.69-0.88)		0.74 (0.67-0.81)		0.49 (0.38-0.61)		0.48 (0.40-0.55)
**Signal properties**									
	Total power in 0.5-10 Hz		0.80 (0.69-0.91)		0.77 (0.70-0.85)		0.74 (0.60-0.89)		0.72 (0.61-0.82)
	Cadence		0.55 (0.43-0.66)		0.52 (0.44-0.60)		0.41 (0.32-0.50)		0.45 (0.38-0.52)
	Width of dominant peak		0.51 (0.45-0.58)		0.50 (0.45-0.55)		0.71 (0.63-0.78)		0.66 (0.60-0.73)
	Total power and width		0.83 (0.73-0.93)		0.78 (0.71-0.85)		0.76 (0.62-0.90)		0.72 (0.61-0.82)

^a^PD: Parkinson disease.

^b^AUC: area under the receiver operator curve.

### Comparison of Patients and Controls

The width of the dominant peak differed between patients and controls in most sensor locations, both premedication and postmedication (Figure 3). Interestingly, the PD patients’ width was elevated in the ankle, pants, and lower back sensors but lowered in the wrist of the most affected side. The estimated cadence did not differ between patients (pre- and postmedication) and controls, regardless of the sensor location used. The total power and height of the dominant peak were lower in patients with PD (premedication) compared with controls in most sensor locations, but this difference was not present after medication intake, with the exception of the wrist of the least affected side, which showed elevated values after medication intake and no differences before medication intake. The ROCs for the various sensor locations and signal properties (Figure 4) show that it was generally more difficult to discriminate between the gait of patients with PD and controls than between before and after medication intake within patients with PD. The AUC and balanced accuracy of all locations and signal properties are presented in Table 3.

The patients’ decision values obtained by the classifier based on the ankle of the most affected side demonstrated low to moderate correlations with the time since diagnosis (ρ=0.55, 95% CI 0.11 to 0.78), the absolute subtotal of the MDS-UPDRS part III mobility items (ρ=0.41, 95% CI –0.07 to 0.81), and the absolute TUG score (ρ=0.33, 95% CI –0.16 to 0.69).

### Impact of Real-world Factors

We observed considerable variation in the premedication and postmedication classification performance (AUC) between individual patients (Multimedia Appendix 1). This may be partly explained by variation in the strength of the response to dopaminergic medication. In addition, real-world factors unrelated to PD may negatively impact the classification performance in uncontrolled settings. By inspecting the video recordings and PSDs, we identified various factors that may have influenced the PSD of devices worn on the wrist and in the pants pocket.

#### Wrist-Worn Devices

**Figure 5:**
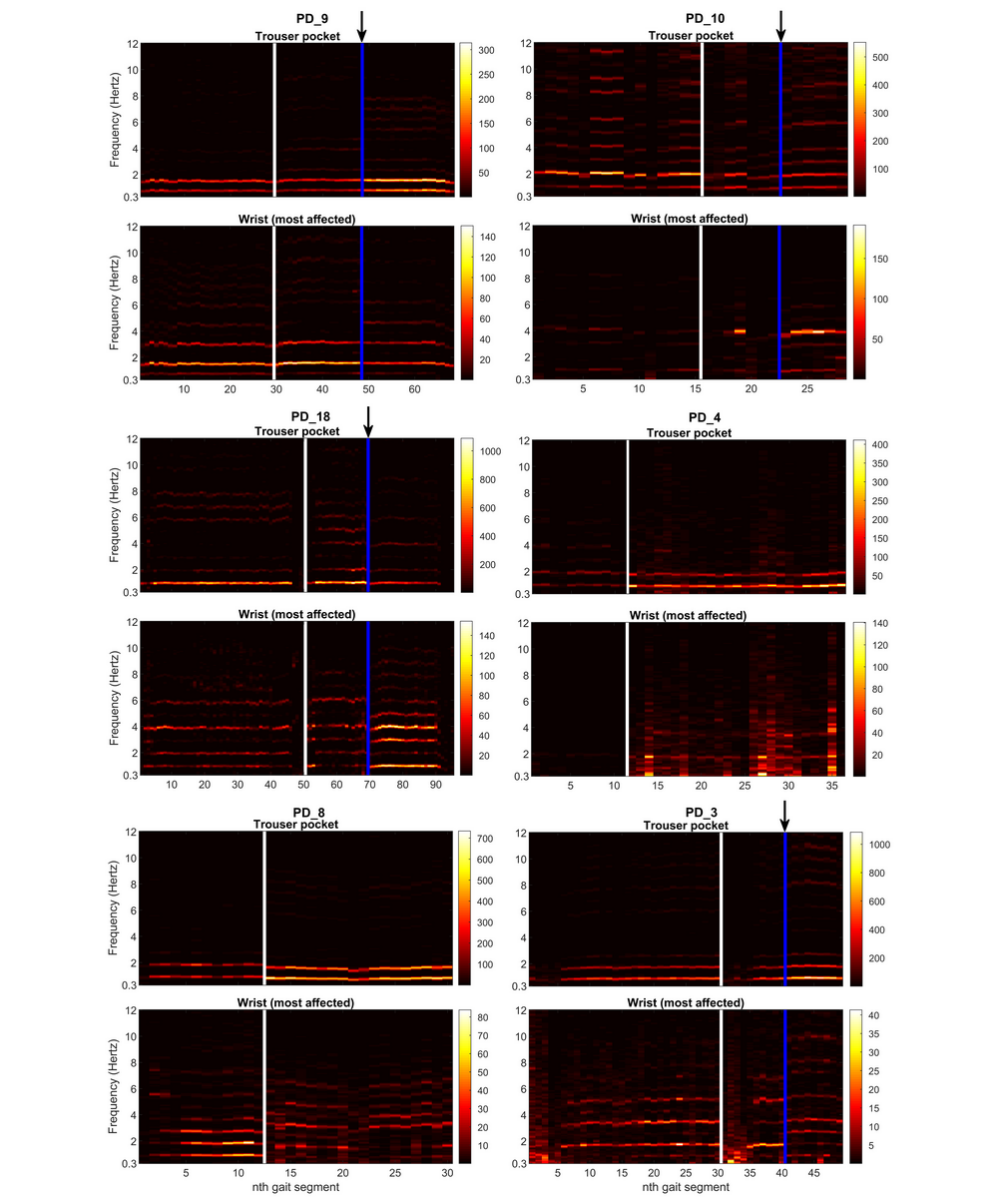
Visualization of the PSD of the devices worn on the wrist (most affected side) and in the pants pocket of 6 illustrative patients. x-axis: nth gait segment of 25 seconds (the white line indicates intake of dopaminergic medication). y-axis: frequency (hertz). Color bar: PSD in (m/s^2^)^2^/Hz. PD_9: blue line with arrow indicates when patient moved his hand from his pants pocket to the pocket of his jacket (during the premedication part, his hand was in his pants pocket). PD_10: blue line with arrow indicates when patient removed her hand from the pocket of her jacket (during the premedication part, her hand was outside the pocket). PD_18: blue line with arrow indicates when patient put his hand in his pants pocket (during the premedication part, his hand was outside the pocket). PD_4: after medication intake, the patient presented with choreic dyskinesias in both arms and legs, which were most severe on his most affected side. PD_8: before medication intake, the arm swing on his most affected side was practically absent. After medication intake, he showed an arm swing with a small amplitude and started to make occasional gestures. PD_3: before medication intake, the arm swing on his most affected side was practically absent (hand was outside the pocket). After medication intake, first he had his hand in the pocket of his jacket. Blue line with arrow: patient removed his hand from the pocket and demonstrated an arm swing with a small amplitude, and he started to make occasional gestures.

For the wrist-worn devices, the positioning of the hands (eg, in or out of the pocket of the pants or jacket) appeared to have a significant impact on the PSD during gait. To explore this, we evaluated the video recordings and PSDs of all patients (n=5) who changed the position of their hands within either the premedication or postmedication free-living part (to be able to rule out that the observed difference was caused by the effects of medication). In all 5 patients, we found an association in time between changes in the position of the hands and changes in the PSD (examples are shown in Figure 5). One patient (PD_9) changed the position of his hands from the pants pocket to the pocket of his jacket, which was associated with changes in the PSD. In the other 4 patients, the nature of the changes in the PSD was associated with the amplitude of the arm swing; in one patient with a pronounced arm swing (based on the video recordings), the total power clearly increased when removing the hand from the pocket (PD_10). In 3 patients with a reduced arm swing, the contribution from the harmonic frequency components changed without an increase or even with a decrease in the total power (PD_3, PD_11, and PD_18).

The effect of an (almost) absent arm swing in patients with PD may closely resemble the effect of putting the hand in the pants pocket. This is illustrated by patients PD_3 and PD_8 (Figure 5). Before medication intake, their arm swing was practically absent, while after medication intake, these patients showed an arm swing with a small amplitude and started to make occasional gestures (eg, point at things). Similar to the patients who removed their hands from their pockets and demonstrated a modest arm swing, the contribution from the harmonic frequency components changed without an increase in the total power (Figure 5).

The video recordings provided no explanation for the low performance of the wrist-worn sensor (most affected side) in PD_7 and PD_15.

#### Pants Pocket

The positioning of the hands also appeared to be relevant when using the pants pocket as a sensor location. Two patients removed their hand from the pants pocket containing the smartphone during one of the free-living parts (PD_9 and PD_18). In both patients, this was not only reflected in the PSD of the wrist-worn device, but also in the PSD of the smartphone (Figure 5). Given the observed changes, we hypothesize that the presence of the hands in the pants pocket reduces the total power measured by the smartphone, which might be explained by reduced freedom of movement of the smartphone. There were other patients with low classification performance in the smartphone (PD_10 and PD_15), but the video recordings provided no explanation.

## Discussion

### Principal Findings

This work presents a new reference data set for the development of wearable sensor algorithms that can passively monitor persons with Parkinson disease in daily life. The novelty of this data set consists of (1) the inclusion of unscripted daily activities in and around the participants’ own homes in combination with a simultaneous video reference that was later used to annotate specific activities and symptoms, (2) the combination of multiple movement sensors positioned on 6 different body locations and 8 different contextual and physiological sensors (eg, GPS, light, photoplethysmogram), (3) objective monitoring of these everyday activities during both a practically defined off state and a subjective optimal on state, allowing for testing of responsiveness, (4) the addition of a subsequent longitudinal follow-up with a more limited set of sensors on 2 body positions for 2 weeks, and (5) the principle of data sharing, such that this rich and versatile data set will be made openly available to the scientific community.

We have also explored this data set ourselves, with the main aim of assessing whether analysis of free-living gait can be used to obtain a digital biomarker for motor fluctuations in PD. We demonstrated that, despite the natural variation that is inevitably present in unscripted gait, it is feasible to measure the response to dopaminergic medication using simple signal properties derived from the PSD of accelerometer signals. Moreover, we present the first simultaneous evaluation of multiple sensor locations in this context. This is a highly relevant issue in the field of wearable sensors, where much uncertainty exists about the ideal sensor placement to obtain the most sensitive and reliable markers for any specific sign of PD [26]. Our results indicate that the effects of dopaminergic medication on gait can be detected in all tested sensor locations (ie, both ankles, both wrists, the lower back, and the pants pocket). Combining multiple sensor locations did not improve classification significantly, suggesting that 1 individual sensor is a reasonable setup, from both an algorithmic and a usability perspective, to monitor on-off changes in free-living gait. Based on the used signal properties, it was generally more difficult to distinguish between patients with PD and age-matched controls than between premedication and postmedication among patients with PD. This may be explained by the fact that, in addition to the presence of PD, other factors also influence a person’s gait pattern (eg, idiosyncrasies, age, comorbidities). When studying within-subject changes, many of these factors remain stable and hence do not introduce variability that is unrelated to PD. Finally, the qualitative analysis using the video recordings and PSDs revealed that the positioning of the hands has a significant impact on the PSD of both the wrist and the pants pocket in real-world settings, highlighting potential points of improvement for future research. Note that we illustrate this approach here for gait, but the data set lends itself well to future analyses by other groups who may want to study the optimal composition of sensor types and positions for a wide range of other symptoms or unscripted daily activities.

### Limitations

Before sensor-based gait analysis can be recommended to clinicians and researchers as a tool to assess therapy responses in real life, it is important to consider this study’s limitations. First, we only included patients with at least some motor fluctuations *and* PD-related gait impairments. Although motor fluctuators may also be the main target group for such an objective evaluation tool, the generalizability to fluctuators who do not report any gait impairments remains to be evaluated. Second, differences in gait pattern between on and off periods might be less pronounced in daily life because most patients do not withhold their medication for as long as they did in this study. We purposely included a practically defined off assessment (ie, after overnight withdrawal of all dopaminergic medication) to assess which sensor locations and signal properties would be most responsive. Nevertheless, our findings remain to be confirmed on truly real-life data, where fluctuations can be more subtle. We should also note that, for practical reasons, we could not randomize the order of the premedication and postmedication parts (the premedication session was always performed first), so we cannot rule out that fatigue influenced the measured postmedication performance (although we minimized its effects by including a break between both parts). Third, the presented assessment of motor fluctuations depends on the occurrence of gait segments of at least 25 seconds. This limits the potential target group to patients who are able to walk during both on and off periods, excluding patients with Hoehn & Yahr stage 5. However, most fluctuators with a lower Hoehn & Yahr stage can still walk when the medication effects have worn off [37]. Finally, the presented analysis depends on accurate localization of gait segments in time. In this study, we used the available video annotations for this, which enabled us to focus on how well the different sensor locations capture on-off changes in the gait patterns without introducing additional variability in how well the different sensor locations can detect gait. Several methods have been proposed for the detection of gait based on accelerometer data, with accuracies varying across sensor locations and techniques [38,39]. We are currently working on the implementation and evaluation of a novel probabilistic gait segmentation framework, which localizes gait segments in time with stationary (periodic) behavior [40].

### Comparison With Other Strategies

In contrast to the analysis of a specific behavior, such as gait, others have proposed activity-independent algorithms to monitor motor fluctuations in daily life. These algorithms typically assume that whenever the patient is wearing the sensor, it is possible to estimate the disease state. For example, Hammerla et al [8] used deep learning to predict the patient’s motor state (ie, asleep, off, on without dyskinesias, or on with dyskinesias) every 5 minutes based on the accelerometer signal from 2 wrist-worn devices. The resulting performance was suboptimal (sensitivity for off detection: 0.50; for on detection: 0.52). This suggests that it may be too ambitious to predict the patients’ motor state regardless of the activities performed.

Another consideration is the interpretability of such activity-independent algorithms. Because it is unclear what the algorithms’ predictions are based on, it is challenging to relate the outcomes to clinical impairments. This might form a barrier to use in clinical practice and makes it difficult to form hypotheses about potential covariates and confounders in daily life. In our approach, it is transparent that the outcomes relate to (changes in) the gait pattern. This means that we can predict circumstances in which the algorithm will fail (eg, when a patient only uses a wheelchair to travel longer distances) and that we can use our knowledge about gait to interpret the results.

### Clinical Interpretation

Our results show that the total power in the 0.5- to 10-Hz interval of the PSD during gait increases in response to dopaminergic medication, whereas the cadence increases in some but decreases in other patients. The width of the dominant peak does not change. The finding related to cadence contrasts with earlier lab-based findings showing an increase in cadence after intake of levodopa [14]. We demonstrated that, because of its marked variability between patients in real life, cadence is not a useful signal property for patient-independent algorithms. A likely explanation for this variability is that the ability to modulate cadence is intact in persons with PD, and some patients use this as compensation for reduced step length [41].

Whereas the clinical meaning of cadence is clear, the clinical meaning of the total power and width of the dominant peak are less straightforward. Sama et al [24] refer to the total power in the 0- to 10-Hz interval as the “fluidity of patients’ movements during gait.” However, the most pronounced clinical changes after medication intake are an increase in step length and arm swing [14,15]. Since it is reasonable to assume that an increased step length and arm swing are reflected in an increased power of acceleration, the total power in the 0.5- to 10-Hz interval is most likely sensitive to these changes in gait pattern. The finding that the total power of the least affected arm is elevated in patients with PD after medication intake in comparison with controls may be explained by excessive movements (including dyskinesias) caused by dopaminergic medication. The width of the dominant peak in the PSD has been referred to as a measure for the inconsistency or variability of gait [21,42]. Weiss et al [21] showed that it predicts the fall risk in patients with PD. Interestingly, although the width of the dominant peak did not change after medication intake in this study, it was elevated in patients with PD compared with controls for the sensors worn on both ankles, the pants pocket, and the lower back. This might be explained by the insensitivity of balance impairments to levodopa [15]. The finding that the width of the dominant peak is lowered in the wrist of the most affected side, pointing to less variability, can possibly be attributed to reduced or absent arm swing and gestures, resulting in a less variable PSD (which matches with our observations, described in “Impact of Real-world Factors”).

We observed absent or weak correlations between the sensor-based predictions and currently used clinical assessments (TUG and MDS-UPDRS part III mobility items). There are multiple explanations for this. The measurement modality is different (for the TUG, it is time versus acceleration-based measures), and the measured construct is related but not identical (for the TUG, postural transitions and gait versus only gait). Perhaps the most important difference is the measurement setting. There is growing evidence that following instructions in a standardized track results in different gait patterns compared with walking freely in a natural environment [16,18-20]. It can be argued that measurements in daily life are more valid, if the aim is to know how the patient performs in the presence of real-life facilitators and challenges. Since free-living assessments are inherently different from clinical assessments, alternative methods to establish validity may be more appropriate. The predictive power (eg, the ability to detect the effect of dopaminergic medication) and, in the end, the clinical utility (eg, whether the information helps clinicians to provide better treatments) might serve as reasonable alternatives.

### Future Directions

The combined inspection of the video recordings and PSDs highlighted some factors other than PD that may impact the (within-subject changes in the) gait pattern in real life, such as putting the hands in the pocket and making gestures. This part of the study was exploratory, and the effects of these factors need to be examined quantitatively in independent experiments. Other potential time-varying factors include voluntary changes in the walking pace [12], the type of shoes worn, carrying objects, the ground type, the location (eg, crowded city versus countryside), and other factors in the physical environment [43]. This area of research has not received much attention so far, largely due to a lack of available data sets. However, controlling for important covariates and confounders could be a significant step forward in the analysis of free-living gait. The inclusion of multiple sensor types that measure context in this study (eg, GPS, Wi-Fi, barometer) and the emergence of smart home sensors [22,44] provide an opportunity to further examine the added value of a multimodal gait pattern analysis. Finally, real-life gait analysis in patients with PD may not only be useful to monitor motor fluctuations but could also yield much-needed digital biomarkers to quantify disease progression in clinical trials testing new disease-modifying therapies. Future studies that include long-term passive monitoring with wearable sensors (such as the Personalized Parkinson Project [45]) will reveal whether the promising results of lab-based gait analysis in this context [12,13] can be further improved by using highly frequent measurements obtained in the patients’ natural environment.

## Appendix

Multimedia Appendix 1 Additional information and analyses.

## Abbreviations

AUC: area under the receiver operator curve
BEAT-PD: Biomarker and Endpoint Assessment to Track Parkinson’s Disease
CV: cross-validation
FOG: freezing of gait
LASSO: least absolute shrinkage and selection operator
MDS-UPDRS: Movement Disorders Society Unified Parkinson’s Disease Rating Scale
PD: Parkinson disease
PSD: power spectral density
ROC: receiver operator curve
TUG: timed up and go test
