# Supplementary material for: Real-Life Gait Performance as a Digital Biomarker for Motor Fluctuations: The Parkinson@Home Validation Study
Source: J Med Internet Res. 2020 Oct 9;22(10):e19068. doi: 10.2196/19068 (PMC7584982; doi:10.2196/19068)
Supplement: Multimedia Appendix 1 [file jmir_v22i10e19068_app1.pdf]

Multimedia Appendix

A: Flow diagram of screening and inclusion

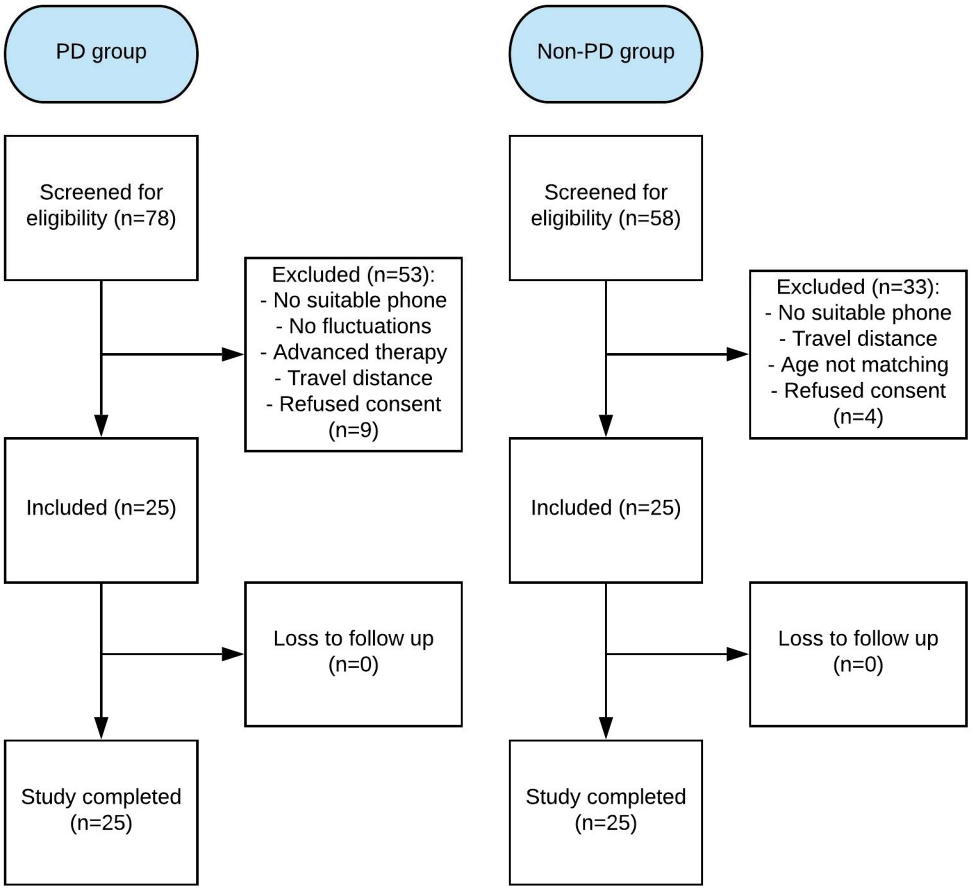

Figure 1: Flow diagram of the screening and inclusion procedure. Not having a suitable smartphone was a reason for exclusion (alone or in combination with other exclusion criteria) in 30 out of 136 screened candidates. Other common reasons for exclusion were the absence of motor fluctuations (score of 0 on the MDS-UPDRS item 4.3, in 19 out of 78 screened patients), and too long travel distance from the hospital (exact numbers were not tracked).

B: Overview of the activities during the study visits

Table 1: Overview of the study visit of the PD group. During the post-med clinical assessment, part I and IV of the MDS-UPDRS were conducted first, to ensure that the MDS-UPDRS part III, TUG and AIMS were performed at least 30 minutes after medication intake. Duration is an indication and may vary between patients. MDS-UPDRS: Movement Disorder Society-sponsored revision of the Unified Parkinson's Disease Rating Scale. TUG: Timed Up-and-Go test. AIMS: Abnormal Involuntary Movement Scale.

Duration (min)	Protocol part	
**0:00-0:15**	Explanation and preparation of sensors	
**0:15-0:45**	MDS-UPDRS part III, TUG and AIMS (pre-med)	
**0:45-1:45**	Free living part (pre-med)	
**1:45-2:00**	Medication intake	Evaluation of motor state of every 30-minute epoch (assessor and patient)
**2:00-3:00**	Complete MDS-UPDRS, TUG and AIMS (post-med)	
**3:00-4:00**	Free living part (post-med)	
**4:00-4:30**	Completion and preparations for study follow-up	

Table 2: Overview of the wearable sensors used during the study visits. Android smartphone: various models with the HopkinsPD app collecting raw sensor data. Android Wear smartwatch: Motorola Moto 360 Sport with custom application collecting raw sensor data. PPG: photoplethysmogram. GSR: galvanic skin response. GPS: global positioning system.

Device	Locations	Collected sensor data
**Gait-up Physilog 4**	Both ankles<br>Both wrists<br>Lower back (strap around waist)	Accelerometer<br>Gyroscope<br>Magnetometer<br>Barometer
**Android Wear smartwatch**	Wrist (PD group: most affected side (self-reported), control group: most comfortable side)	Accelerometer<br>Gyroscope<br>Barometer<br>Light
**Android smartphone**	Trouser pocket (same side as Android Wear smartwatch)	Accelerometer<br>Magnetometer<br>Light<br>Proximity<br>GPS<br>WiFi & cellular networks
**Empatica E4**	Wrist (other side than Android Wear smartwatch)	GSR<br>PPG<br>Skin temperature<br>Accelerometer

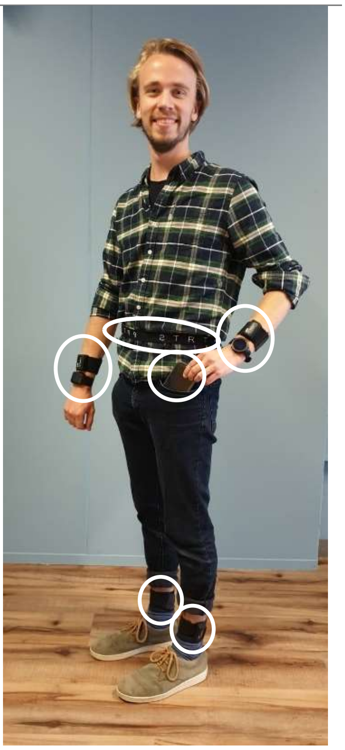

C: Data collection during 2-week follow-up

The Parkinson@home Validation study included a 2-week follow-up period, which started directly after the home visit. Participants were asked to wear a smartwatch and their own smartphone (in the trouser pocket whenever possible) during daytime, which continuously collected raw sensor data in the background (table 1). In addition, participants completed various paper diaries. The daily diary included fall incidents (both groups), intake of PD medication, and freezing of gait (PD group only). The detailed diary was completed for two days for every 30-min epoch, and assessed daily life activities using open text fields (both groups) and motor fluctuations using the Hauser diary and custom questions about tremor and gait performance (PD group). After the 2-week period, participants completed an online survey, which included the self-reported items of part I and II of the MDS-UPDRS, the New Freezing of Gait Questionnaire (NFOG-Q), the fall history, the Short Questionnaire to Assess Health-enhancing Physical Activity (SQUASH) and questions about how subjects experienced the trial. In addition, all participants received a telephone call to assess relevant comorbidities affecting the participant's movements during the study.

Table 3: Overview of the data collection during the 2-week follow-up period. PPG: photoplethysmogram. GSR: galvanic skin response. GPS: Global positioning system. Android smartphone: HopkinsPD app collected raw sensor data. Android Wear smartwatch: custom application collecting raw sensor data.

Device	Locations	Collected sensor data
Android Wear smartwatch	Wrist (PD group: most affected side, control group: most comfortable side)	Accelerometer<br>Gyroscope<br>Barometer<br>Light
Android smartphone	Trouser pocket (same side as Android Wear smartwatch)	Accelerometer<br>Magnetometer<br>Light<br>Proximity<br>GPS<br>WiFi & cellular networks

D: Video annotation protocol

Overview

Objective: The complete home-visits of the Parkinson@home validation study were recorded on video. Because this is considered as personal data, the raw video recordings will not be distributed and only be stored on the internal servers of the study sponsor (Radboudumc). However, participants in the study have provided consent for sharing the video annotations with researchers who have been granted permission from the Michael J Fox Foundation to use the data for research purposes. The goal of this protocol is to create ground truth annotations which can be used as a reference for the sensor data obtained during the home visit.

Protocol: Firstly, the videos are annotated by a research assistant, who creates all the annotations that do not require any clinical expertise. The research assistant creates single annotations for the following domains:

1. General protocol structure: this will allow researchers to separate the free-living measurements from the standardized assessments, and to locate the synchronization markers in the video.
2. Mobility states during free living: these annotations will provide a reference for algorithms that aim to detect behavior during the free parts of the protocol. With the focus on analyzing general behavior (such as walking, sit-to-stand) instead of specific activities (e.g. doing the dishes), we have annotated the overall "mobility states" only (e.g. walking, sitting, stairclimbing, standing up, etc.).
3. Timestamps clinical tests motor examination: this can be used by researchers who aim to quantify the UPDRS part III motor tasks using wearable sensors. The annotations will allow researchers to locate the tasks in time. The reference for the participant's performance on these tasks consists of (1) the scores from the assessor present during the home visit, and (2) the scores from a second rater (clinical expert), who provides additional scores for the UPDRS part III tasks based on the video recordings (see next paragraph).
4. Medication intake and 30 min evaluation of the motor status: this information is already captured in the CRFs from the home visit by both the patient and assessor. The research assistant only transfers these annotations to the video annotations (and check the timestamps noted during the home visit).
5. Falls and near-falls episodes: any fall-related incident is labelled, although (near-)fall episodes are expected to be rare during the home visit.

After this, each participant's UPDRS part III tasks (both in ON and OFF) is re-rated based on the video recordings, by a physician with experience in movement disorders (note: only for the tasks that can be rated using only video). In addition to this, each video is annotated by 1 trained research assistant, who labels the occurrence and type of any freezing of gait episodes and tremor during the free-living parts of the protocol. These annotations are checked by a physician with experience in movement disorders.

Set-up: software called "ELAN" is used, which is an open source program for creating annotations in video recordings (https://tla.mpi.nl/tools/tla-tools/elan/). ELAN is installed on a research PC running within the internal network of the Radboudumc. A template is available with all the different annotations trails and labels. ELAN supports exporting all annotations and corresponding timestamps in a standardized format to .txt files.

Protocol

To be performed by trained research assistant

1. General protocol structure

Please identify all protocol parts performed in the home visit and place the correct labels throughout the video. You can use the notation time present in the CRF as an indication for the start and end times. Make sure the different protocol parts are adjacent (the end time of the previous part is equal to the start time of the next part).

Use the activities described below to identify the parts in the video and place the labels accordingly:

- 1. Start synchronization of sensors:
    - Start: exact frame of first impact
    - End: exact frame of last impact
- 2. Installation of sensors;
    - End: moment when all sensors are worn by participant
- 3. Motor examination (in OFF state);
    - End: end of last motor examination task
- 4. Free living part 1 (in OFF state);
    - End: beginning of questionnaires
- 5. Questionnaires
    - End: beginning of instructions for first motor examination task
- 6. Motor examination (in ON state)
    - End: end of last motor examination task
- 7. Free living part 2 (in ON state)
    - End: moment when first sensor is taken off
- 8. Taking off sensors;
    - End: exact frame of first impact synchronization
- 9. End synchronization of sensors;
    - End: exact frame of last impact

2. Mobility states during free living parts and questionnaires

Participants performed a variety of activities throughout the home visit. In this study, we are interested in labelling the main "mobility states" from the participants, e.g. whether they are walking, sitting, standing, etc.[1,2]. Please annotate the mobility states as listed below during the free living and questionnaire parts of the recordings, and make sure all annotations are adjacent (end of the previous behavior is the start of the next behavior).

- 1. Sitting: when the participant is in a sitting position (the body is in contact with the seat and the upper body is upright and at a +/- 90º angle in relation to the legs);
    - Start: the moment when the body makes contact with the seat.
    - End: when the participant starts a transition to another posture.
- 2. Standing: when the participant is in an upright position with no displacement or with no more than 5 distinctive steps;
    - Start: when the participant's body reached the upright position.
    - End: when the participant starts a transition to another position or starts walking.
- 3. Walking: any episode in which the participant performs 5 or more consecutive steps. A step is defined as the period from the moment when the heel of one foot is off the floor until the same foot makes full contact with the floor;
    - Start: when the participant takes the first step (moment when the heel lifts off the floor)
    - End: when the participant completes the last step (moment when the foot makes full contact with the floor)
- 4. Turning: when the participant makes a turn of at least 90º.
    - Start: the initiation of the first step of the turn (moment when the heel lifts off the floor)
    - End: when the participant completes the last step of the turn (moment when the foot makes full contact with the floor)
- 5. Stair climbing: when the participant is walking up- or downstairs (discriminate between the two in the annotations).

  5.1 walking upstairs

  5.2 walking downstairs
    - Start: initiation of the first step of the climb (moment when the heel lifts off the floor)
    - End: when the participant completes the last step of the climb (moment when the foot makes full contact with the floor)
- 6. Laying: when the participant is in a horizontal position and either the side, front or the back of the body makes full contact with a surface;
    - Start: when the participant's body reaches the horizontal position.
    - End: when the participant starts a transition to another posture.

- 7. Postural transitions: when the participant is transitioning between sitting, standing or laying (time between end point of previous behavior and start of next behavior). Please annotate using the following categories:
  - 7.1 Sit-to-stand (normal chair)
  - 7.2 Stand-to-sit (normal chair)
  - 7.3 Sit-to-stand (low chair/couch)
  - 7.4 Stand-to-sit (low chair/couch)
  - 7.5 Lie-to-stand
  - 7.6 Stand-to-lie
  - 7.7 Sit-to-lie
  - 7.8 Lie-to-sit
- 8. Exercising on a crosstrainer: when the participant is exercising on a crosstrainer.
  - Start: the initiation of the first movement of stepping onto the crosstrainer
  - End: the moment when the participant is standing again
- 9. Cycling: when the participant is cycling
  - Start: the initiation of the first movement of stepping onto the bike
  - End: the moment when the participant is standing again
- 10. Running: When the participant is running
  - Start: when the participant takes the first step (moment when the heel lifts off the floor)
  - End: when the participant completes the last step (moment when the foot makes full contact with the floor) and is standing or walking
- 11. Driving a motorized scooter: when participant is using a motorized scooter
  - Start: the moment when the body makes contact with the seat of the scooter
  - End: when the participant initiates standing up from the scooter
- 12. Driving a car
  - Start: the initiation of the first movement of stepping into the vehicle
  - End: when the participant is standing or walking outside of the vehicle
- 13. Doing push-up exercises
  - Start: the initiation of moving into the position to do push-up exercises
  - End: the moment when the participant is standing upright or walking
- 99. Unknown: it is not clear which label currently applies because of insufficient quality of the video recordings.

3. Clinical tests during motor examination parts

Please annotate the start and end times of the following UPDRS part III tasks:

- 1. Finger tapping (3.4): label left and right separately

  1.1 Left hand

  1.2 Right hand

  - Start: first finger tapping movement AFTER instructions
  - End: end of last finger tapping movement
- 2. Opening and closing of the hands (3.5): label left and right separately

  2.1 Left hand

  2.2 Right hand

  - Start: first hand movement AFTER instructions
  - End: end of last hand movement
- 3. Pronation supination of the hands (3.6): label left and right separately

  3.1 Left hand

  3.2 Right hand

  - Start: first hand movement AFTER instructions
  - End: end of last hand movement
- 4. Toe tapping (3.7): label left and right separately

  4.1 Left toe

  4.2 Right toe

  - Start: first toe tapping movement AFTER instructions
  - End: end of last toe tapping movement
- 5. Leg agility (3.8): label left and right separately

  5.1 Left leg

  5.2 Right leg

  - Start: first leg movement AFTER instructions
  - End: end of last leg movement
- 6. Arise from chair (3.9): label each trial separately
  - Start: first movement that initiates standing up
  - End: body is in upright position or participant is sitting (again) and stops attempt (when trial fails)
- 7. Walking pattern/TUG (3.10): label each trial separately, and label clockwise or anti-clockwise

  7.1: Clockwise

  7.2: Anti-clockwise

  - Start: Start: first movement that initiates standing up
  - End: participant is sitting in the chair again (resting on the seat)

- 8. Postural stability (3.12): label each trial separately
    - Start: 3 seconds BEFORE retropulsion is applied
    - End: when participant completely recovered his balance and is standing in place again
- 9. Postural tremor hands (3.15): label left and right separately

  9.1 Left hand

  9.2 Right hand
    - Start: arms & hands are in appropriate position (stretched, palms down, fingers apart from each other)
    - End: participants changes arm position (end of trial)
- 10. Kinetic tremor hands (3.16): label left and right separately

  10.1 Left hand

  10.2 Right hand
    - Start: start of first finger-to-nose movement
    - End: end of last finger-to-nose movement

4. Medication intake and motor status of the patient: ON and OFF

Please annotate the time of medication intake (you could use the time in the CRF as an indication) and for each half hours that follows, annotate the patient's and assessor's evaluation of the patient's motor state as indicated in the CRF (again, the times in the CRFs can be used as an indication for placing the timestamps).

- 1. Medication intake
- 2. ON state
- 3. OFF state

Background information: ON is the typical functional state when patients are receiving medication and have a good response. OFF is the typical functional state when patients have a poor response in spite of taking medications. In this protocol, since our participants took the last medication on the evening of the previous day, the period from the beginning of the visit until the first medication intake of the day can be classified as an OFF period. After medication intake, the Hauser diary is used to classify each following half-hour as an OFF or ON period by both the patient and assessor.

5. Falls and near-falls episodes

If the assessors noted any (near-)falls during the visit, the full video will be annotated for (near-)falls. From all other participants, several video fragments when (near-)falls are likely to occur (e.g. when walking indoors around obstacles, turning) will be screened for the occurrence of (near-)falls. In this, special attention will be given to participants with Hoehn&Yahr stage >2 (who did not pass the pull

test). If after a minimum of 15 minutes of watching several sections (both before and after medication intake) no (near-)falls have been observed, it is assumed no (near-)falls occurred. If any (near-)falls are observed during the screening, the full video will be annotated for the presence of (near-)falls.

Please mark the beginning and the end of any visible fall or near fall episode present in the video.

- 1. Fall incident: a fall is defined as an event which results in a person coming to rest inadvertently on the ground or floor or other lower level[3].
    - Start: when the participant's center of gravity height starts to change.
    - End: when the participant is laying on the ground or another lower level.
- 2. Near fall: a near fall is considered as a stumble or loss of balance that would result in a fall if sufficient recovery mechanisms were not activated[4]. Recovery mechanisms could be holding furniture or a person, leaning against a wall or a furniture or relying on a walking aid.
    - Start: when the participant's center of gravity height starts to change.
    - End: when the recovery mechanism is used.

To be performed by trained research assistant & clinical expert

1. Freezing of gait (FOG)

The trained research assistant will be presented with the video recordings from identified walking and standing segments during both free living parts and the questionnaire part (annotated from the complete video by the research assistant). If the assessors noted any FOG during the visit, the full video will be annotated for FOG. From all other participants, several video fragments when freezing is likely to occur (e.g. when walking indoors around obstacles, turning, initiating walking) will be screened for the occurrence of FOG. In this, special attention will be given to participants scoring >0 on any of the UPDRS items related to freezing of gait (3.11, 2.13). If after a minimum of 15 minutes of watching several sections (also before/after medication intake) no FOG has been observed, it is assumed that there is no FOG and the video will not be annotated for this symptom. If any FOG is detected, the full video will be annotated for FOG. A physician with experience in movement disorders will be available for support in case of doubt.

Please mark the beginning and end of any visible Freezing of Gait (FOG) episode, independent of type and manifestation. For this study, a FOG is considered as an unintentional and temporary phenomenon where the feet failed to progress[5]. In case various episodes occur together and it is hard to distinguish the beginning and end, please rate them as one episode.

Please also label the type and manifestation of each FOG episode:

In this trial, we consider types of FOG as[6]:

- 1. Start hesitation;
- 2. Turn hesitation;
- 3. Narrow passage hesitation;
- 4. Destination hesitation;
- 5. Open space hesitation;
- 6. Type unclear

Additionally, manifestation of FOG include[6]:

- 1. Shuffling with small steps;
- 2. Trembling in place;
- 3. Complete akinesia.
- 4. Manifestation unclear

All FOG episodes annotated by the trained research assistant will be checked by a physician with experience in movement disorders. Also, in participants where FOG was annotated, and in participants where no FOG was annotated but who scored >0 on any of the UPDRS items related to freezing of gait (3.11, 2.13), a minimum of 15 minutes of fragments that have not been annotated for FOG will be checked (fragments when freezing is likely to occur, e.g. when walking indoors around obstacles, turning, initiating walking). In case the clinical expert discovers any systematic errors, all annotations for the complete visit will be corrected accordingly.

2. Tremor

The trained research assistant will be presented with the video recordings from both free living parts and the questionnaire part. From all participants, several video fragments when the patient is sitting down will be screened for the occurrence of tremor on the upper and lower extremity of the most affected side according to the MDS-UPDRS part IIII off (items related to postural and rest tremor). If after a minimum of 15 minutes of watching several fragments (both before/after medication intake) no tremor has occurred, it is assumed that there is no tremor, and the video will NOT be annotated for this symptom. If any tremor is detected, the full video will be annotated for tremor. A physician with experience in movement disorders will be available for support in case of doubt.

Please annotate tremor as listed below and make sure all annotations are adjacent (end of the previous segment is the start of the next segment). Tremor is defined as a rhythmic and oscillatory movement of a body part with a relatively constant frequency and variable amplitude. Although tremor can be further classified into resting, postural, action and intention tremors, it can be challenging to

discriminate between those during free-living recordings. Therefore, we will not specify the category of tremor in this protocol. Annotate the most affected side only (determined from MDS-UPDRS part IIII off, items related to postural and rest tremor), separately for the upper and lower extremity. Start a new segment when there is a clear contrast visible, either because the tremor start/stops or the severity abruptly increases/decreases. In case of more gradually increasing/decreasing severity, annotate it as one segment and label the most severe amplitude present during the whole segment.

Use the following definitions:

- 99. Not assessable for more than 3 consecutive seconds because participant is not within view of camera or the quality of the recording is insufficient (e.g. camera is moving to much).
- 98. Significant activity of hand/arm for more than 3 consecutive seconds, no tremor visible.
- 97: Significant activity of hand/arm for more than 3 consecutive seconds, still tremor visible.
- 96: Significant periodic activity of hand/arm for more than 3 consecutive seconds, with similar frequency to tremor.
- If video recording quality is sufficient (no 99) AND no significant activity of hand/arm for more than 3 seconds (no 98/97/96):
  - 0. Normal: no tremor visible
  - 1. Slight or mild: Tremor with amplitude <3 cm
  - 2. Moderate: Tremor with amplitude 3-10 cm
  - 3. Severe: Tremor with amplitude >10 cm

The labels are clarified in this flow chart:

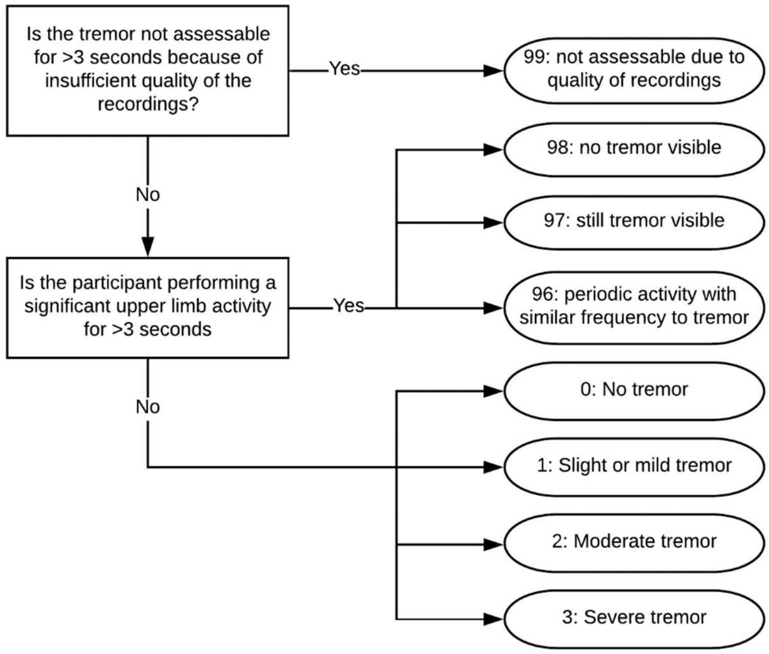

Figure 2: Flow chart of the tremor annotations.

In participants who have been annotated for tremor, a minimum of 50% of all tremor labels will be checked by a physician with experience in movement disorders, with the exception of segments labelled with 99 which will not be checked. In participants who have not been annotated for tremor because the screening was negative, but who scored high on MDS-UPDRS items related to rest and postural tremor (sum of MDS-UPDRS part III off items 3.15a, 3.15b, 3.17a, 3.17b, 3.17c, 3.17d > 6), the screening will be repeated (fragments when the patient is sitting down of minimum 15 minutes will be checked). In case the physician discovers any systematic errors, all annotations for the complete visit will be corrected accordingly.

To be performed by physician

1. MDS-UPDRS part III tasks (1 expert per participant)

The physician with experience in movement disorders will be presented with the video recording segments that belong to the UPDRS part III tasks (annotated from the complete video by the research assistant). Please rate the tasks mentioned below according to the instructions of the MDS-UPDRS (will be provided, together with CRF to note the ratings).

- Finger tapping (3.4)
- Opening and closing of the hands (3.5)
- Pronation supination of the hands (3.6)
- Toe tapping (3.7)
- Leg agility (3.8)
- Arise from chair (3.9)
- Walking pattern/TUG (3.10)
- Freezing (3.11, use video 3.10)
- Postural stability (3.12)
- Posture (3.13, use video 3.10 and 3.12)
- General bradykinesia (3.14, use all earlier video's)
- Postural tremor hands (3.15)
- Kinetic tremor hands (3.16)
- Rest tremor (3.17 & 3.18)

E: Estimation of the participants' cadence

Estimating the cadence (gait cycles/second) from the PSD is often based on the reasonable assumption that the fundamental frequency represents the repetition of gait cycles [1]. However, the fundamental frequency can have a low amplitude because its spectral harmonics (specifically the second and fourth harmonic) are sometimes most prominent, which we most frequently observed in PSD obtained from the wrist and lower back devices. This makes it difficult to reliably and precisely locate the fundamental frequency in some cases. Therefore, we estimated the participant's cadence using the dominant frequency instead, by using one additional assumption that cadence in both PD patients and controls lies within the 0.7 to 1.4 Hz range, which is supported by previous findings [2]. Based on these assumptions, we used a simple rule to derive cadence: if the dominant frequency is below 1.4 Hz, the cadence equals the dominant frequency, if the dominant frequency lies in the 1.4-2.8 Hz interval, the cadence equals half the dominant frequency, and if the dominant frequency is above 2.8 Hz, the cadence equals the dominant frequency divided by four. We should note that we could not empirically validate all estimated cadences because these labels were not available from the video recordings. However, we observed high correlation between the different sensor locations (Spearman's Rho ranged from 0.80 between the two wrist-worn devices to 0.99 between the ankle devices), as would be expected for estimates of cadence.

F: Comparison of using all sensor locations combined vs individual sensor locations

We tested whether the AUCs of the individual sensor locations were lower than the AUC of using all sensor locations combined using the Wilcoxon matched pairs signed rank test ($p<0.05$ considered statistically significant). The results of the individual comparisons were then used to test the overarching null hypothesis that using all sensor locations combined is superior to using any of the sensor locations individually. We rejected this hypothesis if three or more individual comparisons were nonsignificant. The corresponding significance level α corresponds to the chance that we found no significant effect for at least 3 sensors while there was an effect in all individual sensor locations. Given a power of 0.91 to detect a difference in AUC of 0.15 per individual comparison (based on 10.000 simulations of the Wilcoxon signed rank test, assuming a sd of paired differences of 0.20), and assuming independence of the individual comparisons, this chance equals:

$$\alpha = \binom{5}{2} 0.09^3\, 0.91^2 + \binom{5}{1} 0.09^4\, 0.91 + 0.09^5 = 0.0063$$

G: Comparison of PD patients with/without ≥10 gait segments

Table 4: Comparison of clinical characteristics between subjects included or excluded in the analyses (based on whether at least 10 gait segments of 25 sec were available). P-values are based on the Wilcoxon rank sum test (scale variables) and chi-square test (categorical variables).

		**Included (n=18)**	**Excluded (n=7)**	**p-value**
**Time since diagnosis of PD (years)**	Median (IQR)	6.5 (4.8 to 10.3)	8 (5.5 to 10.3)	0.46
**Hoehn & Yahr stage**	Count (%)			0.20
Stage 1		1 (6%)	1 (14%)	
Stage 2		13 (72%)	5 (71%)	
Stage 3		4 (22%)	0 (0%)	
Stage 4		0 (0%)	1 (14%)	
**MDS-UPDRS (scores on subscales)**	Median (IQR)			
Part I		9.5 (7.8 to 15.0)	15 (8.0 to 17.3)	0.41
Part II		11 (8.5 to 15.3)	8 (8.0 to 8.8)	0.15
Part III (off state)		41.5 (31.5 to 57.8)	38 (34.5 to 42.5)	0.65
Part III (on state)		28 (18.5 to 38.0)	30 (20.3 to 37.5)	0.65
Part IV[b]		6 (4.5 to 9.3)	5 (4.3 to 8.3)	0.60

H: Classification results including lower back sensor

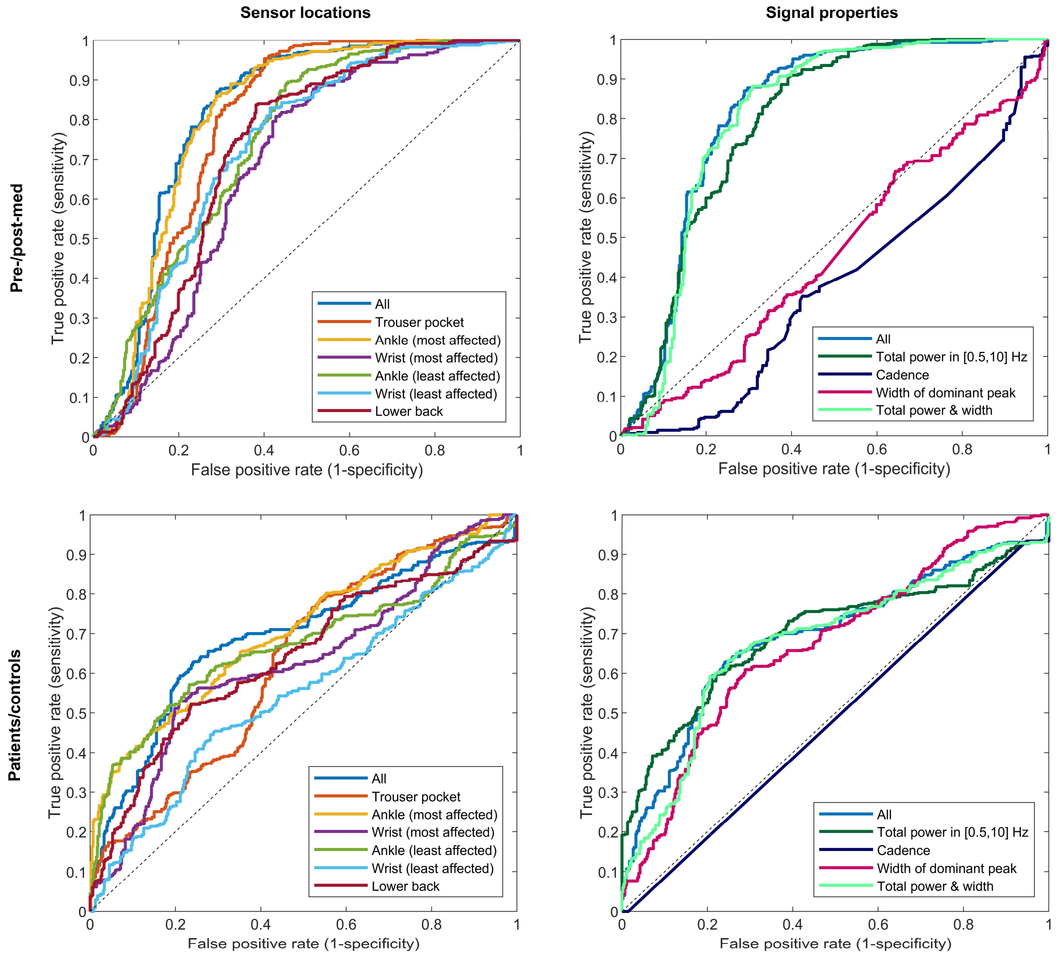

Figure 3: Receiver operating characteristic (ROC) curves of the logistic classifiers, averaged over the cross-validation folds. Top half: pre-/post-med classification (15 patients). Bottom half: patients/controls classification (15 patients and 15 controls). Left half: comparison between different sensor locations. Right half: comparison between different signal properties.

Table 5: Performance of the logistic classifiers (mean ± 1.96 standard error over the cross-validation folds). Accuracies are based on the optimal classifier for each fold with equal misclassification costs and equal class-prior (balanced accuracies). AUC: Area Under the ROC Curve.

**Feature set**	**Pre-/post-med**		**PD patients versus controls**	
	**AUC**	**Accuracy**	**AUC**	**Accuracy**
All	0.82 (0.71 - 0.93)	0.77 (0.69 - 0.86)	0.77 (0.60 - 0.94)	0.69 (0.56 - 0.81)
**Sensor locations**				
Pants pocket	0.78 (0.66 - 0.90)	0.74 (0.65 - 0.83)	0.68 (0.47 - 0.88)	0.59 (0.42 - 0.77)
Ankle (most affected)	0.82 (0.71 - 0.93)	0.78 (0.70 - 0.85)	0.76 (0.60 - 0.93)	0.64 (0.51 - 0.77)
Wrist (most affected)	0.72 (0.60 - 0.83)	0.69 (0.60 - 0.77)	0.68 (0.51 - 0.86)	0.55 (0.42 - 0.68)
Ankle (least affected)	0.71 (0.60 - 0.83)	0.69 (0.59 - 0.78)	0.74 (0.60 - 0.87)	0.65 (0.53 - 0.77)
Wrist (least affected)	0.76 (0.65 - 0.87)	0.70 (0.62 - 0.77)	0.61 (0.45 - 0.77)	0.53 (0.41 - 0.64)
Lower back	0.72 (0.60 - 0.85)	0.71 (0.63 - 0.79)	0.65 (0.47 - 0.83)	0.64 (0.53 - 0.74)
**Signal properties**				
Total power in 0.5-10 Hz	0.77 (0.64 - 0.90)	0.75 (0.66 - 0.83)	0.77 (0.58 - 0.96)	0.67 (0.54 - 0.80)
Cadence	0.40 (0.30 - 0.51)	0.43 (0.36 - 0.50)	0.47 (0.40 - 0.53)	0.49 (0.48 - 0.51)
Width of dominant peak	0.46 (0.38 - 0.53)	0.47 (0.41 - 0.52)	0.72 (0.66 - 0.78)	0.64 (0.58 - 0.69)
Total power & width	0.81 (0.70 - 0.92)	0.78 (0.70 - 0.86)	0.78 (0.61 - 0.95)	0.68 (0.56 - 0.80)

I: Distribution of recording & gait segment durations during the home visits

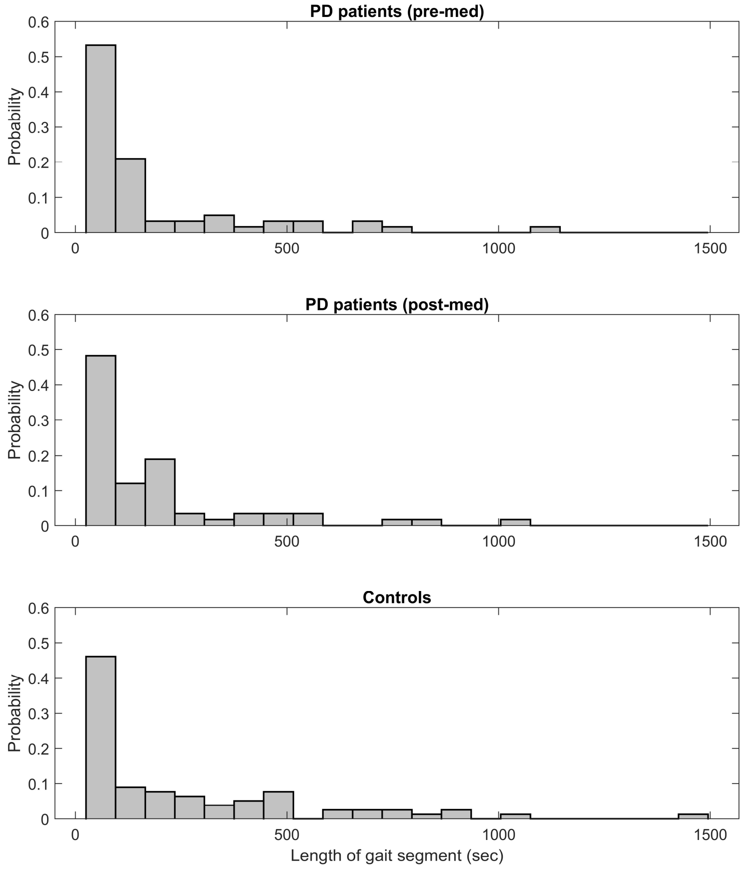

Figure 4: Distribution of the length of all gait segments during the home visits included in the analyses (gait segment length of at least 25 sec). The high overlap indicates that in our dataset, the observed differences between pre-/post-med and PD/controls cannot be explained by differences in gait segment length. In addition, we did not observe any significant correlations between each patient's median gait segment length during the home visits and clinical parameters (time since diagnosis, MDS-UPDRS part III score, and MDS-UPDRS part III mobility items subtotal), both pre- and post-med.

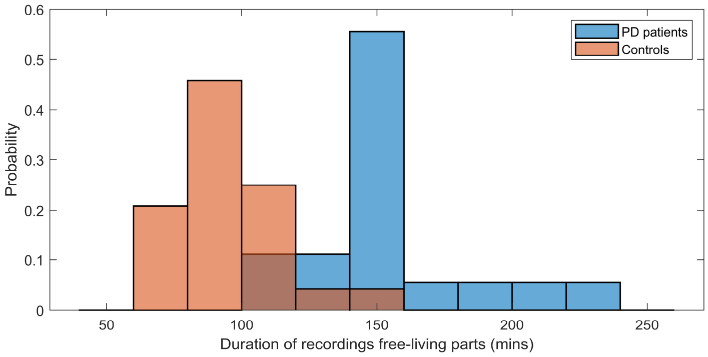

Figure 5: Distribution of the duration of recordings during the free-living parts.

J: Home visit: experiences of PD patients

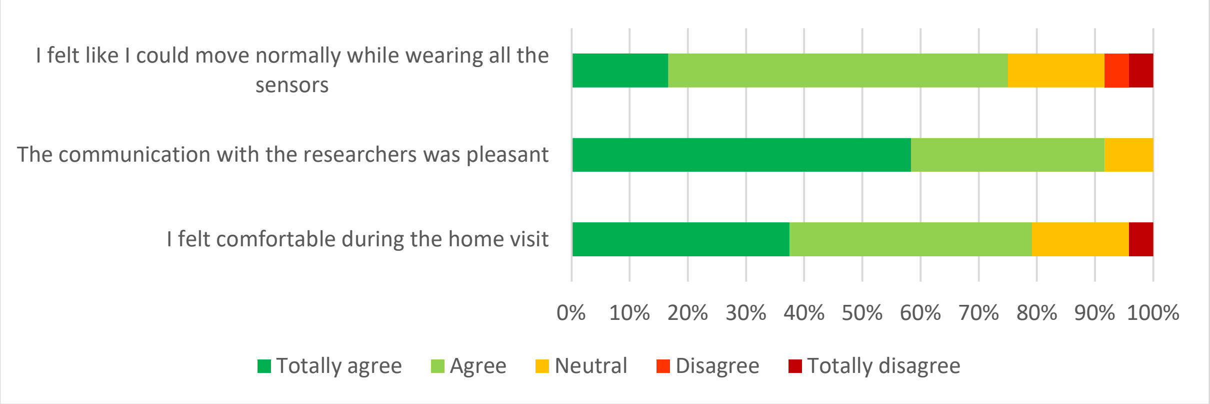

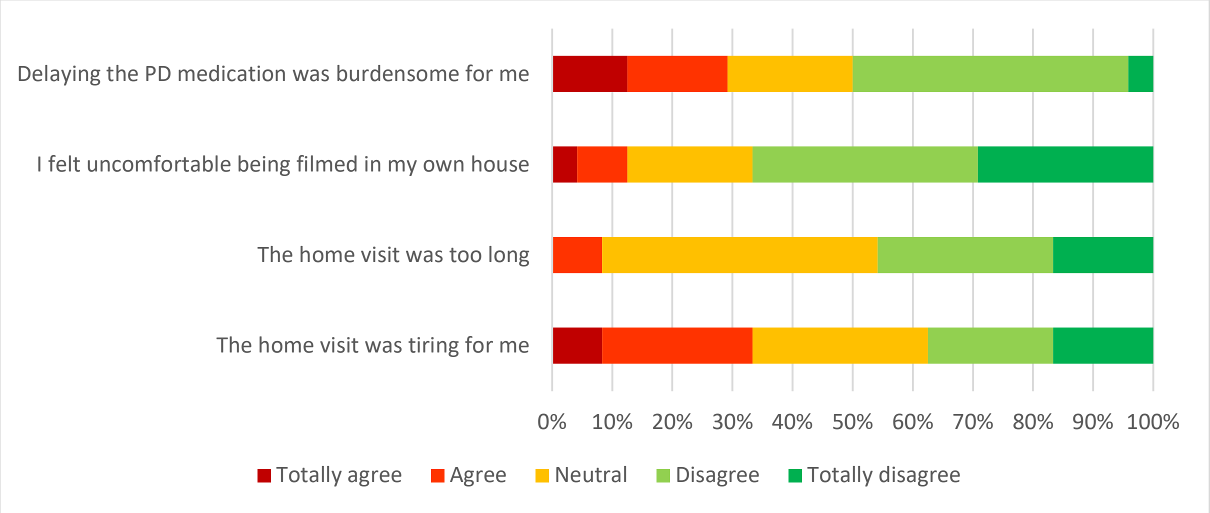

Figure 6: Experiences of PD patients during the home visit, based on an online exit survey (n=24, one survey missing).

K: Sensitivity analysis pre-/post-med classification

Table 6: Performance of the logistic classifiers (mean ± 1.96 standard error over the cross-validation folds). Accuracies are based on the optimal classifier for each fold with equal misclassification costs and equal class-prior (also referred to as balanced accuracies). AUC: Area Under the ROC Curve. Left: data from each subject is centered on each subject's mean, but not scaled by each subject's standard deviation (for the purpose of regularization, data is scaled by each feature's between-subject standard deviation instead). Right: data is not normalized per subject (for the purpose of regularization, data is scaled by each feature's between-subject standard deviation and overall mean instead). These results indicate that centering on each subject's mean is more important than scaling by each subject's standard deviation (without centering data on each subject's mean, using patient-independent thresholds is less successful, as reflected in the drop in accuracy).

**Feature set**	**Centering on mean only**		**No normalization per subject**	
	**AUC**	**Accuracy**	**AUC**	**Accuracy**
All	0.80 (0.69 - 0.90)	0.74 (0.65 - 0.84)	0.74 (0.63 - 0.86)	0.61 (0.54 - 0.68)
**Sensor locations**				
Pants pocket	0.72 (0.61 - 0.84)	0.69 (0.59 - 0.79)	0.70 (0.59 - 0.82)	0.58 (0.50 - 0.66)
Ankle (most affected)	0.80 (0.70 - 0.90)	0.75 (0.66 - 0.85)	0.78 (0.68 - 0.88)	0.60 (0.53 - 0.67)
Wrist (most affected)	0.76 (0.66 - 0.87)	0.71 (0.61 - 0.80)	0.76 (0.66 - 0.87)	0.62 (0.54 - 0.70)
Ankle (least affected)	0.74 (0.64 - 0.83)	0.69 (0.59 - 0.78)	0.71 (0.61 - 0.81)	0.59 (0.51 - 0.67)
Wrist (least affected)	0.80 (0.71 - 0.89)	0.75 (0.68 - 0.83)	0.79 (0.69 - 0.89)	0.62 (0.55 - 0.69)
**Signal properties**				
Total power in 0.5-10 Hz	0.77 (0.65 - 0.88)	0.73 (0.62 - 0.84)	0.75 (0.63 - 0.87)	0.60 (0.53 - 0.68)
Cadence	0.60 (0.48 - 0.73)	0.59 (0.47 - 0.71)	0.51 (0.40 - 0.63)	0.50 (0.44 - 0.57)
Width of dominant peak	0.45 (0.40 - 0.50)	0.47 (0.44 - 0.50)	0.44 (0.39 - 0.49)	0.47 (0.44 - 0.50)
Total power & width	0.78 (0.67 - 0.90)	0.74 (0.64 - 0.84)	0.75 (0.63 - 0.86)	0.63 (0.56 - 0.71)

L: Individual classification performance

Table 7: Area Under the ROC Curve (AUC) of the pre-/post-med logistic classifiers of each individual patient and sensor location (AUCs lower than 0.5 in bold).

	**Trouser pocket**	**Ankle (most affected)**	**Wrist (most affected)**	**Ankle (least affected)**	**Wrist (least affected)**
**PD_1**	0.93	0.91	0.97	0.76	1.00
**PD_2**	0.90	0.94	1.00	0.92	0.96
**PD_3**	0.64	0.84	0.59	0.64	0.73
**PD_4**	0.98	1.00	1.00	0.99	0.96
**PD_5**	0.94	1.00	0.76	**0.37**	0.89
**PD_6**	0.73	0.81	0.84	0.75	0.80
**PD_7**	0.62	0.68	**0.42**	0.76	0.71
**PD_8**	1.00	**0.10**	**0.49**	**0.29**	0.77
**PD_9**	0.81	0.98	**0.36**	0.91	**0.28**
**PD_10**	**0.44**	0.86	0.83	**0.48**	**0.41**
**PD_11**	0.83	0.68	0.69	0.82	0.59
**PD_12**	0.86	1.00	1.00	1.00	1.00
**PD_13**	0.93	0.84	0.75	0.85	0.93
**PD_14**	0.96	0.95	0.97	0.96	0.82
**PD_15**	**0.13**	0.85	**0.37**	0.70	0.62
**PD_16**	0.92	0.83	0.86	0.58	0.90
**PD_17**	0.93	0.59	0.99	0.57	0.90
**PD_18**	**0.46**	0.92	0.83	0.89	0.95

References

1. Fasel, B., et al. Medical & biological engineering & computing, 2017. **55**(10): p. 1773-1785.
2. Ambrus, M., J. Sanchez, and M. Fernandez-Del-Olmo. Gait & posture, 2019. **68**: p. 136-140.
